# Regime shifts in South Florida groundwater: distinguishing upstream recharge from coastal sea level influences (1997–2022)

**DOI:** 10.1007/s10661-026-16000-x

**Published:** 2026-10-08

**Authors:** Ethiopia B. Zeleke, Assefa M. Melesse, René M. Price, Tania Islam, Shimelis Dessu, Theresa Strazisar, Steven Krupa, Alexandra Hoffart, Fred Sklar

**Affiliations:** 1 Department of Earth and Environment, Institute of Environment, Florida International University, Miami, FL 33199 USA; 2 South Florida Water Management District, West Palm Beach, FL USA

**Keywords:** Florida Bay, Freshwater discharge, Spatiotemporal variability, Groundwater, Temporal regime shifts

## Abstract

Groundwater dynamics in South Florida play an important role in shaping the hydrologic balance of the region. This study investigated long-term trends in groundwater elevation, surface water stages, and their interactions across Water Conservation Areas 3A and 3B, Everglades National Park, coastal watersheds, and Florida Bay. Time series decomposition of long-term groundwater elevation (1997 to 2022) was used to identify trends, seasonal patterns, and anomalies. Analysis of groundwater elevations revealed statistically significant regime shifts defining distinct phases of decrease (2005–2015) and increase (2016–2022) associated with changes in rainfall patterns and water management. Average groundwater elevation has increased in recent years, with upstream areas showing stronger associations with surface water flow volumes, while coastal areas showed stronger correlations with sea level changes. This distinction suggests separate hydrological mechanisms for the observed trends: upstream recharge likely dominates in freshwater marshes, whereas coastal areas are associated with sea level variability. Groundwater levels in coastal areas now remain above the ground surface even during dry periods, associated with external factors such as sea level rise. The relationships between surface water stages and groundwater elevations in most parts of the study area were linear, whereas coastal regions, parts of the Water Conservation Areas 3A and 3B and northern parts of the Taylor Slough were nonlinear. In Florida Bay, sea level variability was a dominant driver of surface water variability (88.3% relative importance), while freshwater delivery through creek discharge (2.2%) and rainfall (2.6%) showed small but measurable contributions, along with groundwater elevation (2.4%) reflecting regional hydrologic conditions.

## Introduction

Groundwater is a vital freshwater resource for agriculture, drinking water, human health, and ecosystem function. Owing to rapid population growth, there is a growing demand for groundwater resources (Lee et al., 2012). Satellite observations reveal that groundwater storage is rapidly decreasing in many parts of the world (Richey et al., 2015; Rodell et al., 2009). Studying the spatial and temporal variations in groundwater elevations enables evaluation and prediction of groundwater responses to anthropogenic events and climate change (Taylor et al., 2013). Factors such as groundwater withdrawal, hydrological characteristics, and shifts in rainfall patterns can contribute to the spatial variability of groundwater storage (Swain et al., 2019). Understanding these spatial patterns guides the development of site-specific monitoring and management strategies.

South Florida is characterized by flat topography and a wide platform of porous carbonate rocks that facilitate surface-groundwater interactions. In the Everglades, surface water, sheet flow, sloughs, canals, and standing water across inundated marsh overlie groundwater in the unconfined surficial aquifer systems (SAS). The uppermost unit of the SAS, the Biscayne Aquifer, is a highly porous, exceptionally transmissive karst aquifer (Fish & Stewart, 1991; Price et al., 2003). Surface water and shallow groundwater are continuously exchanged across the wetland surface through recharge and discharge (Harvey et al., 2004a, 2004b), and in many areas, their levels effectively coincide, confirming a close hydraulic connection (Price et al., 2003). Where the karst lies at or near the surface and the marsh is flooded, water can rise above the land surface so that surface water and the shallow water table become effectively continuous (Price et al., 2003). Where a thicker, low-permeability peat layer intervenes, vertical head gradients develop that govern the magnitude and direction of exchange (Harvey et al., 2004a, 2004b). Although hydraulically coupled, surface water and groundwater may differ in flow rate, residence time, and chemistry. The degree to which the two coincide or diverge is governed by water-control infrastructure and natural features, including stratigraphic variability, meteorologic processes, seasonality, and saltwater intrusion from tidal water bodies (Carter et al., 2010; Harvey & McCormick, 2009; Prinos et al., 2014; Swain et al., 2019). South Florida groundwater is particularly vulnerable to anthropogenic water use and diversion, sea level rise, and saltwater intrusion (Price et al., 2006), with the region experiencing expansion of agricultural activities and groundwater withdrawal (Blanco et al., 2013). Management of the water conveyance system in South Florida has had a significant effect on fluctuations in surface water and groundwater. Historical changes in hydrology, such as extensive drainage from 1880 onward and the construction of major canals between 1910 and 1920, have significantly altered surface water flows (F. Sklar et al., 2002; F. H. Sklar et al., 2019). The construction of levees and borrow canals has contributed to changes in surface flows, which have disrupted hydroperiods and the long-standing balance between freshwater and saltwater (F. Sklar et al., 1999). By lowering water tables, drainage has exposed the organic peat soils to oxygen and accelerated their decomposition, lowering the land surface through subsidence (F. Sklar et al., 1999; Stephens & Johnson, 1951). Altered hydrology and nutrient enrichment have likewise favored the expansion of cattail into sawgrass marshes and sloughs, particularly along canals (Newman et al., 1998). Together, these changes upset the balance between peat accumulation and loss that maintains wetland elevation (Parker et al., 1955; Sklar et al., 1999).

Florida Bay is a large shallow bay located south of and adjacent to the Florida Peninsula. The substantial hydrological modifications within the peninsula of South Florida have decreased surface water and groundwater flows into Florida Bay (Fourqurean & Robblee, 1999; Light & Dineen, 1994; Sklar et al., 1999). Modifications to the freshwater inflow into Florida Bay started in 1950 and due to the altered hydrologic conditions of the region led to notable changes over time (Sullivan et al., 2014). Alterations to Taylor Slough (TS) through canal construction have disrupted natural freshwater inputs into Florida Bay and instead redirected water toward the Atlantic Ocean. Such hydrological changes manifested as elevated salinity in the Bay, saltwater intrusion into freshwater marshes (Ross et al., 2000), and development of hypersalinity and die-off of benthic vegetation (Fourqurean & Robblee, 1999). The ecological consequences of these modifications became apparent through recurring environmental crises. The 1987 seagrass die-off, followed by ecosystem effects in sponges, shrimp, and lobster fisheries, triggered scientific attention (Butler et al., 1995; Durako et al., 2001; Peterson et al., 2006). Similar ecological challenges resurfaced during the 2014–2015 drought, when hypersalinity conditions again led to seagrass die-offs and subsequent ecosystem disruptions (Hall et al., 2016). These recurring crises highlighted knowledge gaps, particularly regarding groundwater-bay connectivity and salinity relationships.

Efforts to restore historic freshwater inflows from TS began in 1980, with subsequent restoration projects (Sullivan et al., 2014). However, the hydrologic changes induced by canal construction have had lasting impacts on the hydrology of Florida Bay (Sklar et al., 2003). Saltwater intrusion within the shallow surficial aquifer now extends 15–30 km inland of Florida Bay, thereby cutting off fresh groundwater discharge to the Bay (Fitterman & Deszcz-Pan, 1998; Langevin et al., 2005; Price et al., 2006; Saha et al., 2012). In response to these ongoing challenges, initiatives like the Groundwater Exchange Monitoring and Modeling (GEMM) Plan have been developed to address scientific uncertainties. Understanding the interaction between surface water and groundwater remains crucial for effective management and restoration efforts in the region.

The water table in South Florida has been consistently mapped since the 1940 s (Prinos & Dixon, 2016). Early efforts by Cross and Love (1942) documented water levels at different time periods during 1940 and provided a baseline understanding of the region’s hydrology before the implementation of water control structures. Parker et al. (1955) expanded on this by creating detailed water table maps for selected areas in South Florida, illustrating conditions during the initial stages of water management. Subsequent investigations by the United States Geological Survey in 1979 further characterized the surficial aquifer system (Merritt, 1997). Fish and Stewart (1991) detailed the properties of the aquifer system, groundwater flow, and hydraulic responses to natural and anthropogenic perturbations. More recently, Lietz et al. (2003) developed detailed maps showing average water levels over 5-year periods in southeastern Florida. Prinos and Dixon (2016) extended this approach by mapping groundwater elevations across South Florida and conducting statistical analyses to reveal spatial and temporal patterns for the period of 1997 to 2009.

These foundational studies highlight the evolving understanding of groundwater dynamics and the factors driving changes in South Florida. Building on this foundation, the present study aims to provide a comprehensive analysis of the variability of groundwater elevation in the region from 1997 to 2022 and identify the hydrologic factors influencing Florida Bay and its surrounding freshwater marshes. The specific objectives are to:

- Discern short- and long-term temporal patterns and decadal changes in groundwater elevation
- Identify the spatial variability in groundwater elevation among well-groups with shared attributes
- Quantify the influence and timing of upstream natural and modified flows on water levels in Florida Bay

## Study area

**Fig. 1:**
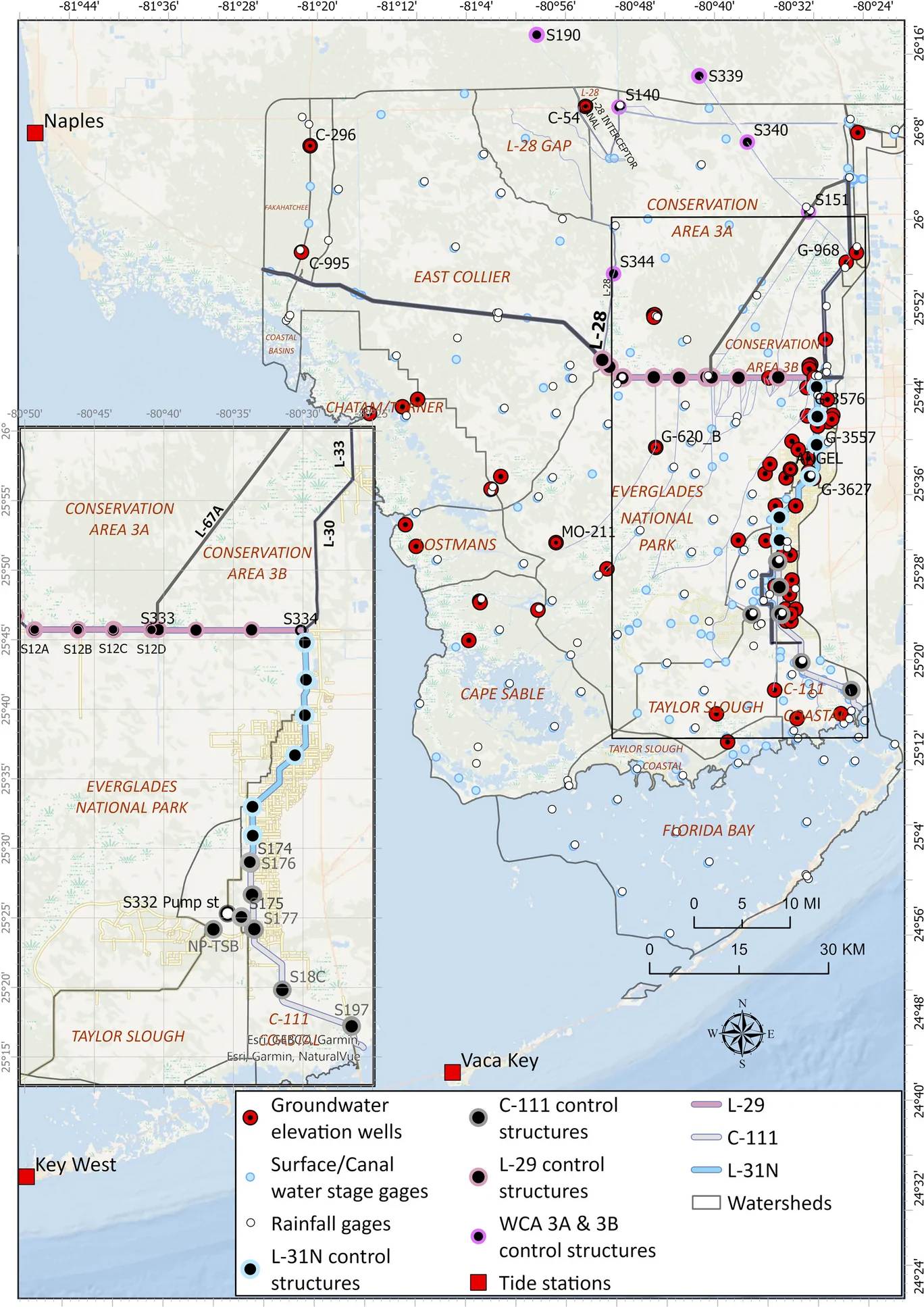
Location of monitoring stations and water control infrastructure across the study area. Groundwater elevation wells, surface water and canal stage gages, and water control structures including L-31N, C-111, and L-29 are shown. Tide stations at Key West, Vaca Key, and Naples provide sea level reference data. Major canals (L-29, C-111, L-31N) and watershed boundaries (gray outlines) delineate the primary hydrologic units including Water Conservation Areas 3A & 3B, Everglades National Park, East Collier, and coastal systems. The inset shows detail of the southern monitoring network of structures along the C-111 canal system extending toward Florida Bay

The study area (Fig. 1) represents the terminal portion of the historic Everglades ecosystem where freshwater flow from upstream areas converges with tidal marine waters. The area covers approximately 13,000 km^2^ (5000 mi^2^) of the hydrologic system of southern Florida, including the Water Conservation Areas 3A and 3B, Everglades National Park, Taylor Slough, East Collier watershed, and coastal systems (Chatham, Lostmans, Cape Sable, Coastal Taylor Slough and C-111) extending to Florida Bay.

The area is characterized by extremely low topographic relief, with elevations ranging from approximately −2 to 15 ft NAVD 88 (Jones & Price, 2007). Water movement is controlled by both natural features, including the underlying limestone geology and seasonal precipitation patterns, and engineering infrastructure such as canals, levees, and water control structures that regulate flow between watershed compartments (Swain et al., 2019). The region experiences a subtropical climate with distinct wet (May–October) and dry (November–April) seasons that drive the natural hydrologic cycle. These seasonal patterns, combined with water management operations, create complex surface water-groundwater interactions that vary spatially across the freshwater marshes, transitional zones, and coastal areas that comprise the study domain (Harvey et al., 2000; 2004b; Langevin et al., 2005).

### Description of selected control structures

Geospatial data of canals and streams, as well as canal system descriptions and physical characteristics from Carter et al. (2010), were used to select control structures that influence surface water flows into each watershed in the study area. A brief description of the selected water control structures for each watershed is provided below.

Canals with significant influence on the study area are shown in Fig. 1. The L-29 (Tamiami) canal facilitates water flow eastward to the C-4 borrow canal. During periods of low flow, water is supplied to the C-4 basin, to meet water supply needs, from adjacent water conservation areas (WCAs) via the South Dade Conveyance System (SDCS). Along with L-30 and L-33, the L-29 borrow canal manages water levels in South Florida by conveying water from WCA-3A via the L-67A canal to the L-29 and intercepting seepage from WCA-3B and conveying it to adjacent basins to maintain surface and groundwater elevations for municipal water supply and environmental needs. On borrow canal L-29, gated spillways in WCA-3A, S-12A through D, release flow southward into Everglades National Park (ENP). Additional flow is delivered to the L-29 Canal and crosses into Northeast Shark River Slough through culverts and bridged openings beneath Tamiami Trail between structures S-333 and S-334.

The L-31 N canal serves as a conduit for delivering water from the WCAs to the C-111 basin in southern Miami-Dade County. This canal is part of the SDCS and contributes to maintaining water levels in that region (Carter et al., 2010). Structures S-174 and S-176 discharge water from L-31N to L-31W and C-111 canals. S-176 also contributes to the flow released eastward into the C-111 agricultural watershed, which recharges Florida City well fields. The S-332 pump station and S-175 on L-31W borrow canal discharge flow to Taylor Slough. Contributions from C-111 discharge into Taylor Slough between structures S-18C and S-197 as well as through S-177. Originating at S-176, the flow within the canal progresses southward, discharging via S-197 into Barnes Sound (Carter et al., 2010). Structures S-140 on L-28, S-190 from the L-28 interceptor and feeder canals, S-344 via sheet flow through the L-28 gap and L-28 tieback levee borrow canal, and S-339 and S-340 (which prevent excess drainage of the WCAs) were also considered in the temporal analysis of groundwater elevation in WCAs 3 A and 3B.

## Data

Daily groundwater elevation and surface water stage data for the period from 1997 to 2022 from all monitoring stations in the study area (Fig. 1) were collected from the United States Geological Survey (USGS; https://waterdata.usgs.gov/nwis), the South Florida Water Management District (SFWMD) DBHYDRO database (https://www.sfwmd.gov/science-data/dbhydro), and the South Florida Information Access—Data Exchange (https://sofia.usgs.gov/exchange/sfl_hydro_data/). The daily time series data were averaged to a monthly timescale.

The total depths of groundwater monitoring wells located in the eastern part of the study area range from 8 to 50 ft below land surface (bls), most of which have high to very high hydraulic conductivity (Fish & Stewart, 1991). Given that surface water-groundwater interactions predominantly occur within shallow aquifers, groundwater elevation wells within the surficial aquifer system (< 50 ft depth) were selected for analysis. Along the eastern boundary of the study area, where the highly permeable upper part of the surficial aquifer system is the Biscayne Aquifer, monitoring wells as deep as 100 ft were also included, as they align with the depth of water supply wells that draw water from the Biscayne Aquifer.

**Table 1:** Summary of the datasets used in this study

Data	Sampling rate		Extent		Source
	Spatial	Temporal	Spatial	Temporal	
Groundwater elevation	GEMM* Station Index	Daily	Study area	1997–2022	Laura A DeCicco et al. (2018); Stachelek (2016) retrieved January 20, 2024
Surface water stage and rainfall	GEMM* Station Index	Daily	Study area	1997–2022	
Canal water stage	GEMM* Station Index	Daily	Control structures on canals	1997–2022	
Canal discharge	Control structures	Daily	Control structures on canals	1997–2022	Stachelek (2016) retrieved July 2024
Tide gauge sea level	Tide stations	Daily	South Florida Coast and Key West	1997–2022	Holgate et al. (2013) retrieved January 20, 2024
Aquifer parameters	Surficial aquifer system	-	South Florida	-	Fish and Stewart (1991); Reese and Wacker (2009) retrieved January 20, 2024
Potential evapotranspiration	EDEN grids	Daily	Greater Everglades	1997–2022	Bellino and Mecikalski (2021) retrieved July 2024
Digital Elevation Model	400 m × 400 m continuous girds	-	Greater Everglades	-	Jones and Price (2007) retrieved July 2024

*Groundwater Exchange Monitoring and Modeling (GEMM) plan database (Strazisar et al., 2024)

Surface water stage and discharge data measured at control structures and levees in canals within and around the study area were collected from DBHYDRO. Daily rain gauge rainfall data for the period from 1997 to 2022 were obtained from all stations in the study area and summarized to monthly time series. Monthly public water use reanalysis data for the period 2000 to 2020 (Luukkonen et al., 2023) by the national watershed boundary (HUC12) and daily potential evapotranspiration data were obtained from the Everglades Depth Estimation Network (EDEN) (https://sofia.usgs.gov/eden/evapotrans.php). Monthly average tide gauge sea level records for Key West and Vaca Key stations (which contain sufficient time series records from 1997 to 2022) were obtained from the Permanent Service for Mean Sea Level (PSMSL), 2024 (“Tide Gauge Data,” https://psmsl.org, retrieved January 20, 2024) (Holgate et al., 2013). Geospatial data of watersheds, canals, and streams were accessed from SFWMD AHED (Arc Hydro Enhanced Database). The data used are summarized in Table 1.

**Table 2:** Monthly percentage changes in groundwater elevation and surface water stage at the WCA 3A and 3B and ENP stations between the three periods (1997–2005, 2006–2015, and 2016–2022)

Months	WCAs 3A and 3B				ENP			
	Change in GW elevation (%)		Change in SW stage (%)		Change in GW elevation (%)		Change in SW stage (%)	
	1997–2005 to 2006–2015	2006–2015 to 2016–2022	1997–2005 to 2006–2015	2006–2015 to 2016–2022	1997–2005 to 2006–2015	2006–2015 to 2016–2022	1997–2005 to 2006–2015	2006–2015 to 2016–2022
Jan	−0.1	5.6	−3.0	2.5	−0.4	15.9	−5.1	5.6
Feb	0.0	8.3	−2.4	3.5	−5.8	21.2	−4.9	14.9
Mar	−1.8	8.6	−2.8	2.6	−12.5	27.4	−9.9	21.8
Apr	−4.6	7.1	−3.0	0.2	−15.4	25.0	−5.0	13.9
May	−5.6	10.3	−2.6	1.4	−15.0	26.9	6.9	−12.5
Jun	−6.0	16.3	−4.3	7.1	−8.9	26.1	4.9	15.2
Jul	−5.5	12.4	−4.4	6.2	1.0	16.8	4.9	5.0
Aug	−6.4	9.1	−4.4	3.8	−4.9	16.6	−2.2	6.9
Sep	−4.9	6.4	−2.7	2.1	−4.6	15.1	−2.3	7.9
Oct	−4.7	6.4	−4.1	3.0	−3.4	12.8	−5.3	9.3
Nov	−3.7	8.4	−4.4	4.2	−4.4	13.4	−4.7	15.1
Dec	−1.0	6.2	−3.3	2.7	−5.4	12.6	−1.0	17.2

## Methods

### Temporal analysis: time series decomposition

#### Long-term trends and seasonality

Time series decomposition was used to analyze groundwater elevation dynamics by separating the data into trend, seasonal, and residual components (Lafare et al., 2016; Patle et al., 2015). The trend component captures long-term changes driven by climate variability, hydrological conditions, and anthropogenic influences. The seasonal component shows periodic recharge and discharge cycles. The seasonal component is also used to evaluate temporal lags and the timing of groundwater responses to rainfall. The residual component highlights short-term variations due to irregular or localized factors.

An additive seasonal-trend decomposition (STL) filter (Eq. 1; Cleveland et al., 1997; Cleveland & Devlin, 1988) was applied to monthly groundwater elevation data via Python “statsmodels.api,” and a 12-month period was selected.

1 $${Y}_{\mathrm{t}}={T}_{\mathrm{t}}+ {S}_{\mathrm{t}}+ {R}_{\mathrm{t}}$$

where ${Y}_{\mathrm{t}}$ is the raw time series at time *t* and where ${T}_{\mathrm{t}}{, S}_{\mathrm{t}}, \mathrm{a}\mathrm{n}\mathrm{d} {R}_{\mathrm{t}}$ represent the trend, seasonal, and residual time series components, respectively. Data gaps were filled with long-term average groundwater elevation data to maintain continuity.

The statistical significance of the trend component was evaluated using modified Mann-Kendall trend test (Hamed & Rao, 1998). The “hamed_rao_modification_test” (Hamed & Rao, 1998) accounts for autocorrelated data. Trends were deemed significant at *p* < 0.05. We similarly analyzed long-term trends and statistical significance of corresponding surface water stage.

The seasonal component of groundwater elevation was compared with the seasonal surface water stage and rainfall to estimate the seasonal correlation and identify temporal lags.

#### Residual mass curve analysis

To identify persistent departures from normal conditions and distinct phases of groundwater elevation change, a residual mass curve analysis was performed. This approach complements the time series decomposition by highlighting cumulative deviations from baseline conditions.

The long-term monthly mean (climatology) was computed by aggregating groundwater elevation data by months across all years (Eq. 2):

2 $$\overline{{GW }_{m}}=\frac{1}{N}\sum_{y=1}^{N}{GW}_{y,m}$$

where N is the number of years = 26 for the period 1997–2022 and ${GW}_{y,m}$ denotes groundwater elevation for month m in year y.

Monthly deviation (*Δ*, Eq. 3) from climatology was computed as:

3 $${\Delta}_{y,m}={GW}_{y,m}-\overline{{GW }_{m}}$$

The residual mass curve value for each month (${C}_{t}$, Eq. 4) was calculated as the cumulative sum of these deviations:

4 $$C_\mathrm{t}=\sum_{i=1}^m\Delta_i\;t=1,\dots,12$$

 

To quantify temporal changes (Eq. 5) in groundwater elevation, we calculated monthly rates of change:

5 $$R_\mathrm{t}=\frac{C_t-C_{t-1}}{{\Delta T}_\mathrm{t}}\;{\Delta T}_\mathrm{t}=1month$$

where *R*_t_ represents the rate of change in groundwater elevation for month m, and ${\Delta T}_{\mathrm{t}}$ equals 1 month.

These rates were also aggregated annually to identify yearly patterns using Eq. 6:

6 $${R}_{annual, y}= \sum_{m=1}^{12}{R}_{t}$$

Major recharge and discharge events were identified using statistical thresholds, with annual rates exceeding the 90th percentile classified as significant recharge periods and those below the 10th percentile as significant discharge periods.

To evaluate hydrological drivers and characterize natural water availability patterns, we constructed residual mass curves for precipitation minus evapotranspiration (P-ET). We similarly analyzed volumetric discharge data to quantify the influence of managed flows on the system. Temporal synchronicity and the rate of change in these residual mass curves were assessed alongside groundwater elevation fluctuations. The degree of association between these variables was quantified using Pearson’s correlation coefficient.

The residual mass curve was also used to identify temporal regime shifts, defined as transitions between periods of sustained increase and decrease. To objectively verify these transitions, we applied the Chow test for temporal breaks (Chow, 1960), specifically focusing on periods associated with water management changes. Temporary drought-related fluctuations were excluded from consideration as a potential regime change point unless the statistical significance persisted beyond the drought period.

### Correlation

The monthly stages measured at control structures described in Sect. “Data” and overland over each watershed from 1997 to 2022 were used to assess the degree of synchronicity of the surface water stages with groundwater elevation. Pearson’s correlation coefficient (Eq. 7) was used to quantify the correlation between surface water and groundwater elevation.

7 $$R=\frac{\sum ({\mathrm{x}}_{\mathrm{i}}-\overline{\mathrm{x} })({\mathrm{y}}_{\mathrm{i}}-\overline{\mathrm{y} })}{\sqrt{{\sum}_{\mathrm{i}=1}^{\mathrm{n}}{({\mathrm{x}}_{\mathrm{i}}-\overline{\mathrm{x} })}^{2}}\sqrt{{\sum}_{\mathrm{i}=1}^{\mathrm{n}}{({\mathrm{y}}_{\mathrm{i}}-\overline{\mathrm{y} })}^{2}}}$$

where ${x}_{\mathrm{i}}$ and ${y}_{\mathrm{i}}$ are the stage and groundwater elevation, respectively. $\overline{x} \mathrm{a}\mathrm{n}\mathrm{d } \overline{y }$ denotes the means of the two variables.

### Random forest regression analysis of water level variability in Florida Bay

To analyze the drivers of surface water stage variability in Florida Bay, we implemented a random forest (RF) regression model using the scikit-learn (version 1.5.1) library in Python (version 3.11.4) (Breiman, 2001). RF was selected for its ability to capture nonlinear relationships between environmental variables without requiring distributional assumptions.

**Fig. 2:**
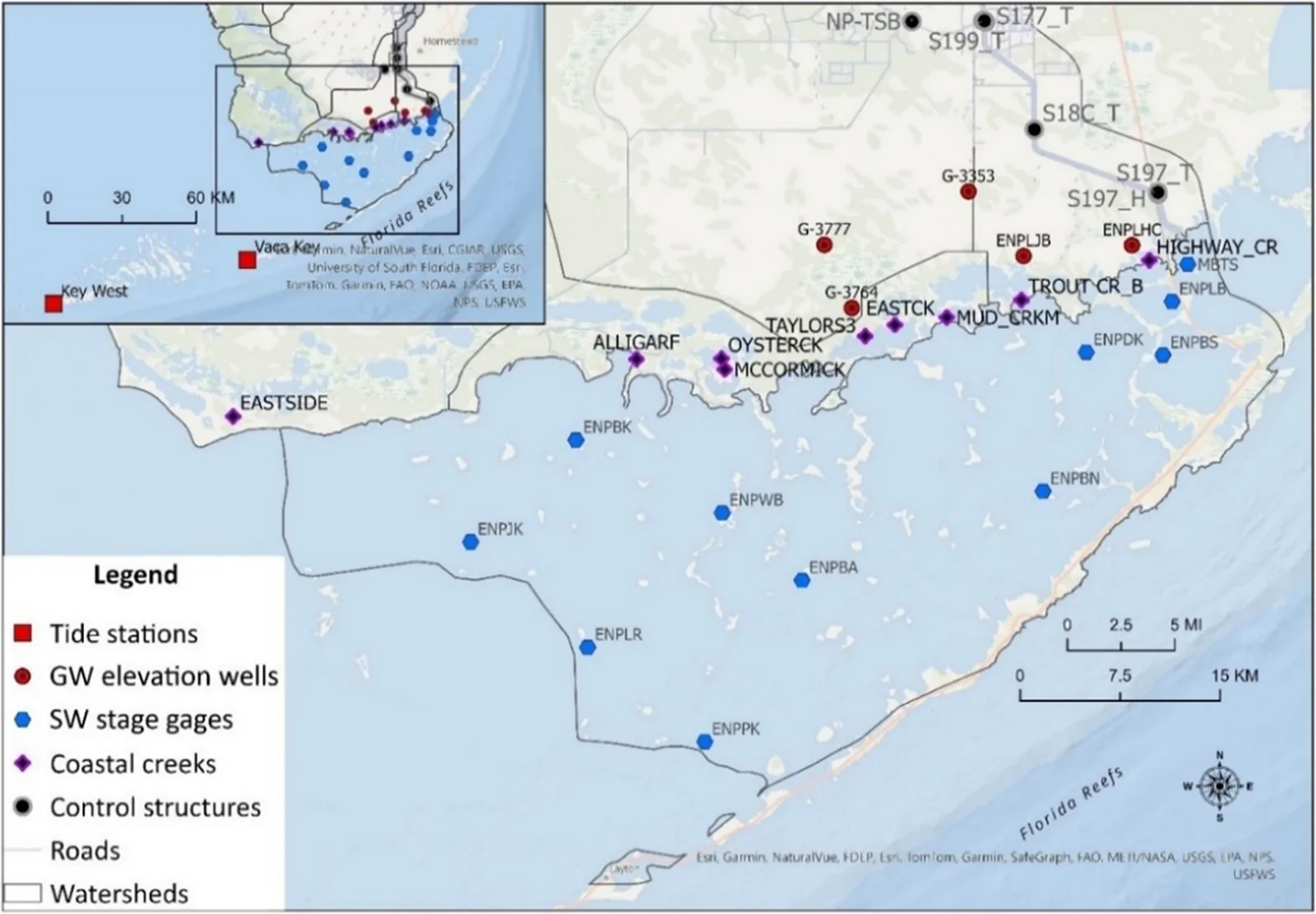
Florida Bay surface water stage gages, coastal groundwater elevation wells, discharge creeks, and tide gages

The model incorporated six predictors: sea level, coastal creek discharge, groundwater elevation in coastal systems (Taylor Slough and C-111 basin), evaporation rates, rainfall, and wind speed (Fig. 2). These factors were chosen on the basis of their well-known interactions and potential to impact stage dynamics in coastal regions (Xin et al., 2022).

Evaporation was estimated using the radiative energy balance approach specified in Price et al. (2007). All variables were processed as monthly time series spanning 2000–2022. 

For temporal validation, we partitioned the dataset chronologically: data from 2000 to 2016 served as the training set, while data from 2017 onward constituted the test set through which model performance was assessed. First-order differencing was applied to both dependent and independent variables to remove trends and seasonality and to maintain stationarity in the time series. 

To evaluate model robustness, we implemented time series cross-validation using scikit-learn TimeSeriesSplit (Pedregosa et al. (2011). Model hyperparameters were optimized through grid search to minimize out-of-bag (OOB) error, an internal performance metric that helps prevent overfitting. 

The relative influence of each predictor on surface water stage was quantified using feature importance scores based on a mean decrease in variance (Breiman, 2001). This metric measures each variable’s contribution to reducing error across all decision trees in the forest and provides hierarchical influence of environmental drivers on surface water stage in Florida Bay.

To ensure model validity, we implemented several statistical diagnostic checks on model residuals. The Shapiro-Wilk test was used to assess normality of residuals, with *p* > 0.05 indicating normally distributed errors. Autocorrelation was evaluated using the Durbin-Watson test, where values approaching 2.0 indicate independence of errors. Homoscedasticity was assessed using the Breusch-Pagan test, with *p* > 0.05 supporting constant variance across predictor values. The augmented Dickey-Fuller test was used to verify stationarity of residuals, with *p* < 0.05 indicating stationarity over time.

### Spatial analyses: clustering and interpolation

Two spatial autocorrelation analyses were performed on groundwater elevation and surface water stage. The first is Moran’s I (Eq. 8), which provides a global measure of the association of a variable and its spatial location and allows for the evaluation of the tendency toward dispersion or agglomeration. The second is a local indicator of spatial association, abbreviated as LISA (Anselin, 1995), which was used for localized well groups and to identify hotspots between high-value well groups and cold spots between low-value well groups as well as potential outliers that do not significantly conform to those well groups. A possible of four statistically significant cluster/outlier patterns can be identified using LISA. These are High-High clusters (well groups) (HH: high groundwater elevation well surrounded by high groundwater elevation wells), Low-Low clusters (well groups) (LL: low groundwater elevation wells surrounded by low groundwater elevation wells), High-Low (high groundwater elevation well surrounded by low groundwater elevation wells), and Low-High (LH: low groundwater elevation wells surrounded by high groundwater elevation wells).

8 $$I=\frac{\mathrm{n}}{\mathrm{W}}\frac{{\sum}_{\mathrm{i}=1}^{\mathrm{n}}{\sum}_{\mathrm{i}=1}^{\mathrm{n}}{\mathrm{w}}_{\mathrm{i}\mathrm{j}}({\mathrm{z}}_{\mathrm{i}}-\overline{\mathrm{z} })({\mathrm{z}}_{\mathrm{j}}-\overline{\mathrm{z} })}{{\sum}_{\mathrm{i}=1}^{\mathrm{n}}{({\mathrm{z}}_{\mathrm{i}}-\overline{\mathrm{z} })}^{2}}$$

where *n* is the total number of observations, *W* is a weight matrix that describes the dependency between location *i* and *j* (Fletcher & Fortin, 2018), and *z* denotes groundwater elevation.

Empirical Bayesian kriging (EBK) interpolation was then applied to the long-term (1997–2022) monthly average groundwater elevations as well as the surface water stages. This spatial interpolation approach uses the spatial dependence of groundwater elevation and surface water stage data to estimate values at unsampled locations. By analyzing semivariogram parameters, optimal weights for neighboring groundwater elevations and surface water stages that minimize variance are determined. The implicit consideration of interdependence among sampling points and uncertainty metrics such as the standard error (SE) of the prediction make kriging interpolation preferable (Lam, 1983). The general form of kriging is shown in Eq. 9.

9 $$z=f\left(x_i\right)+v\left(x_i\right)+\varepsilon_i$$

where *f*(*x*_i_) is a deterministic trend function showing how responses vary with space, *v*(*x*_i_) characterizes spatial dependence through variogram parameters, and *ε*_i_ represents unexplained variations or measurement errors (Fletcher & Fortin, 2018).

To evaluate seasonal variability conditions, the vadose (unsaturated) zone thickness was quantified by calculating the difference between ground surface elevation and groundwater elevation. Ground surface elevations, referenced to NAVD 88, were subtracted from the EBK interpolated groundwater elevations for representative wet and dry months. This analysis provided a spatial assessment of unsaturated zone thickness across the study area and was a means of cross-validating groundwater elevation patterns within the context of actual saturation conditions. Areas with negative or zero values indicated surface water presence or fully saturated conditions with no vadose zone, while positive values represented the thickness of the unsaturated layer.

Hydraulic gradients were also quantified using the Exploratory 3D Analysis toolset in ArcGIS Pro Version 3 (Esri, 2023a). The Elevation Profile function was applied to the EBK interpolated groundwater elevation to calculate gradients along user-defined transects oriented perpendicular to equipotential lines. Gradients were calculated as the change of hydraulic head (∆*h*) divided by distance (∆*l*), expressed in ft/ft. Multiple transects were analyzed within each identified flow direction to establish representative ranges for both May and September conditions, capturing seasonal variability in the hydrologic system.

The spatial relationships between surface water and groundwater elevations were evaluated via the local bivariate relationships tool in ArcGIS Pro Version 3 (Esri, 2023c). This tool quantifies the relationship between two continuous variables across a geographic area by assessing whether the values of one variable depend on or influence the values of the other and how these relationships vary spatially. This method is based on the concept of entropy from information theory, which measures the degree of uncertainty or randomness in a variable and quantifies the mutual information shared between two variables. By estimating joint entropy, the tool identifies statistically significant relationships (Guo, 2010). The analysis utilized surface water stage monitoring stations as spatial reference points for the bivariate relationship assessment. Groundwater elevations were extracted to these station locations from the spatially interpolated groundwater elevation map to allow for a direct comparison between surface water stage and groundwater elevation at consistent spatial coordinates. The analysis was performed via a neighborhood-based approach wherein the data were normalized, spatial neighborhoods were identified, and the relationships were tested for significance. To determine the optimal neighborhood size, we generated distance statistics between monitoring points using the Generate Near Table tool in ArcGIS Pro V.3 (Esri, 2023b). For each potential neighborhood configuration, we calculated the minimum, maximum, and average distance between monitoring points. These distance metrics were evaluated against known hydrological patterns in South Florida to ensure that they would be appropriate for isolating distinct coastal and inland groundwater-surface water interaction regimes. The same neighborhood configurations were also assessed for information content (entropy gain) and statistical significance. The configuration that provided sufficient spatial resolution while maintaining reasonable statistical power was selected.

## Results

### Temporal patterns of groundwater elevation

#### Water Conservation Areas—3A and 3B

Groundwater elevation trends in WCAs 3 A and 3B were analyzed via data from 10 wells, with average elevations ranging from 2 ft NAVD 88 in WCA-3B to 12 ft NAVD 88 at C-54 (L-28 Gap).

**Fig. 3:**
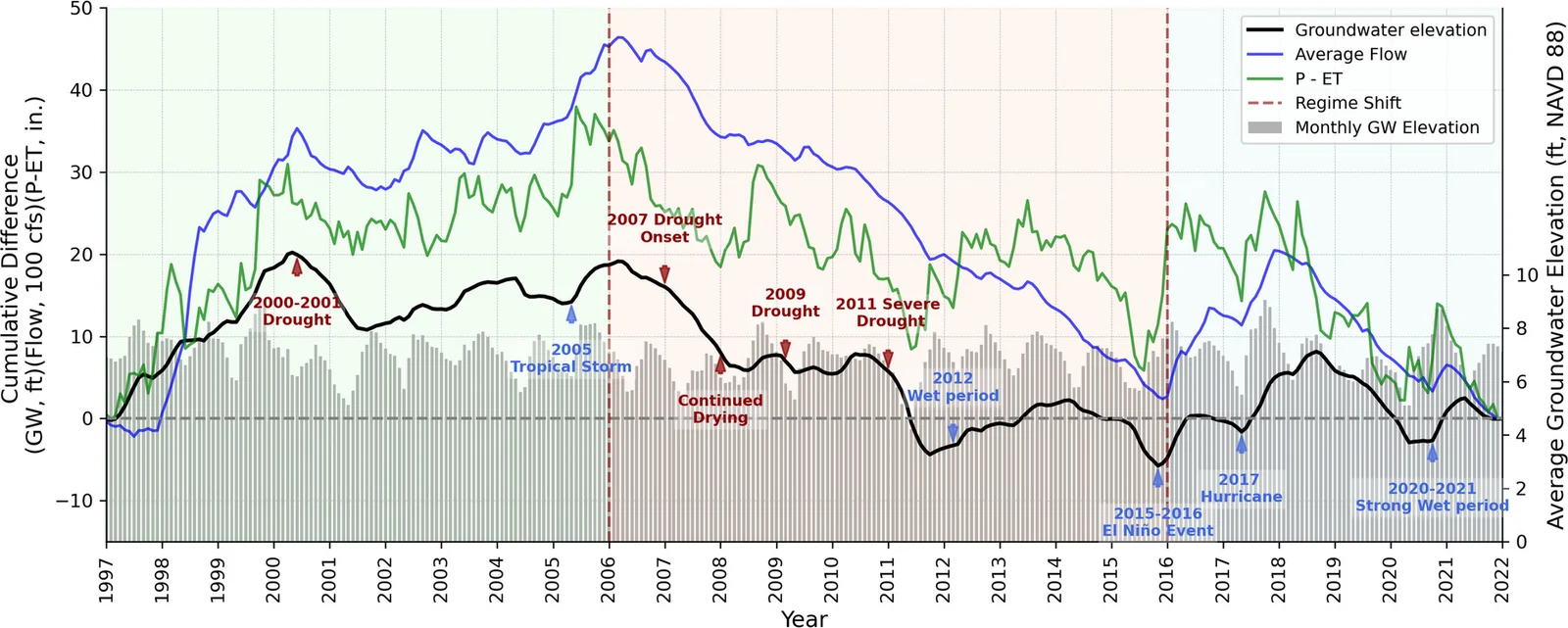
Residual mass curve of hydrological variables in WCA 3A and 3B (1997–2022). The primary *y*-axis shows cumulative differences from monthly means for average groundwater elevation (*n* = 10), average flow, and precipitation minus evapotranspiration (P-ET), while the secondary *y*-axis shows monthly average groundwater elevation (grey bars). Red markers indicate drought events, and blue markers highlight wet periods (drought.gov). Vertical dashed red lines denote temporal regime shifts

Statistical analysis identified two regime shifts in groundwater elevation patterns between 1997 and 2022. The first shift occurred in 2006 (*F* = 71.88, *p* < 0.0001), marking the transition between an accumulation and relatively stable period to a declining phase. The second shift occurred in 2016 (*F* = 8.10, *p* < 0.001), representing the transition to a reversal phase (Fig. 3). These statistically verified regime shifts define three distinct periods: 1997–2005, 2006–2015, and 2016–2022. Between 1997–2005 and 2006–2015, a 6% reduction in mean groundwater elevation was observed during peak wet months (June–August), with 2006–2015 characterized by a reduction (Table 2). In contrast, 2016–2022 showed a reversal, with average groundwater elevation increasing by up to 16% compared with that from 2006 to 2015, particularly in May–July (+10–16%) and December–January (+6%).

The modified Mann‒Kendall trend test indicated a statistically significant increasing trend in four stations. Along the southeastern border of WCA-3B (stations 3BS1-GW2, 3BS1-GW3, and 3BS1-GW4) had an average increase of 0.0384 ft/year during the 2000–2022 period. Additionally, station C-54 in the L28-Gap showed an increase of 0.024 ft/year over the 1997–2018 period.

The surface water stage trends (*n* = 34) resulted in a statistically significant decreasing trend in stations S343_h, S333_H, and S343B_H with an average rate of −0.0263 ft/year during 1994–2022. These stations are found along the L-29. In contrast, stations WCA3ATI9 (2006–2022) and 3BS1W1 (2000–2022) had a statistically significant increasing trend with an average rate of +0.038 ft/year. The remaining stations did not have a statistically significant trend. Evaluation of overall average surface water stages in WCAs 3A and 3B over the regime-shifts defined in Fig. 3 showed that the period 1997–2005 had higher surface water stages followed by 2016–2022. The period 2006–2015 remained low, similar to average groundwater elevation. The declining phase (2006–2015) is punctuated by significant drops in groundwater elevation during 2006–2008, 2009, 2011–2012, and 2015, which directly correspond to documented drought periods in the region. These intervals have a cascading hydrological response: reduced P-ET values trigger decreased average flow and surface water stage, which subsequently manifests as sharp groundwater depletion. Moderate to high correlation was also noted between natural drivers (P-ET) and groundwater elevation where Pearson’s correlation coefficient = 0.618 while higher correlation was observed between managed water release (average flow) and groundwater elevation (*r* = 0.795). This implies a greater degree of association of groundwater elevation in WCAs 3A and 3B to managed flows compared to natural factors.

#### Everglades National Park

Analysis of groundwater elevations was done in 17 Biscayne Aquifer wells in the ENP ranging from 6 ft NAVD 88 (G-3576) to below 0 ft NAVD 88 (MO-211). Statistically significant long-term trends were observed in stations G-3437 (1997–2022) L31NSGW1, L31NSGW2, L31NSGW3, L31NSGW4 (2004–2022) with an average rate of +0.066 ft/year. These stations are found along and near canal L-31N.

**Fig. 4:**
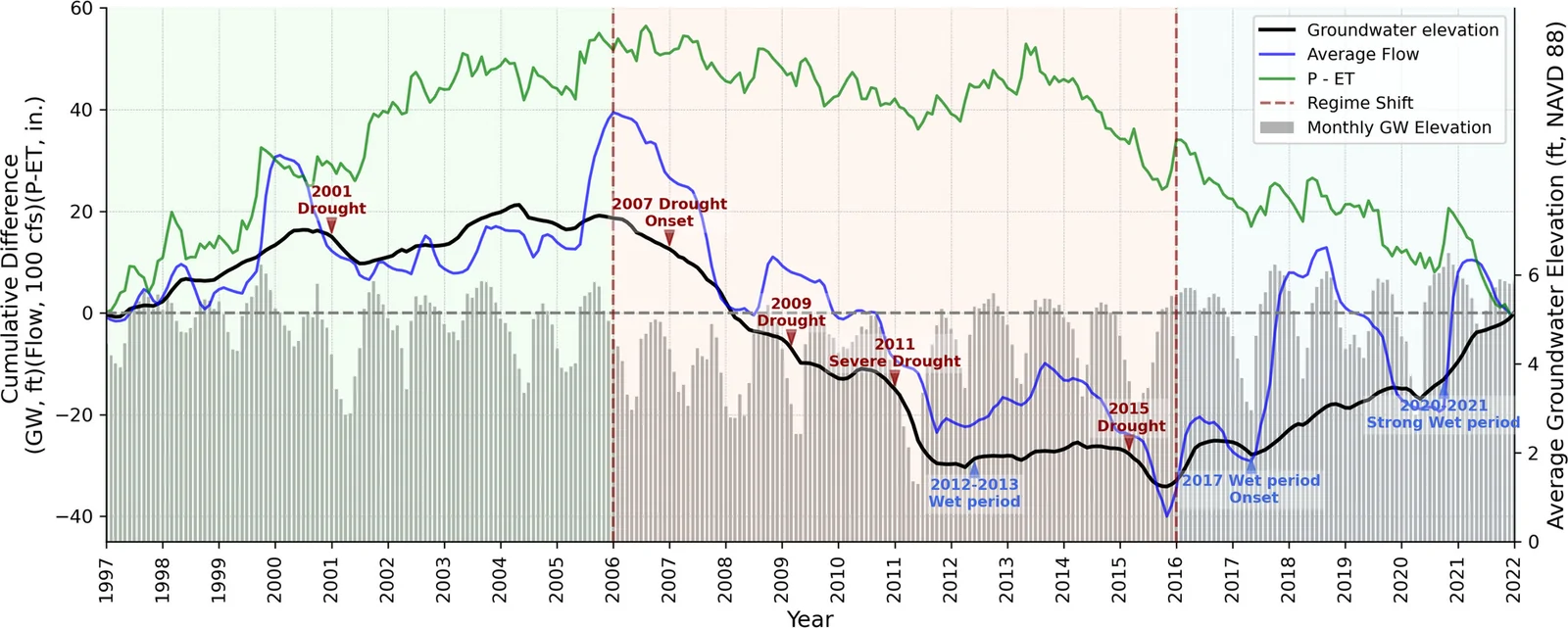
Residual mass curve of hydrological variables in the ENP (1997–2022). The primary *y*-axis shows cumulative differences from monthly means for average groundwater elevation (*n* = 17), average flow, and precipitation minus evapotranspiration (P-ET), while the secondary *y*-axis shows monthly average groundwater elevation (grey bars). Red markers indicate drought events, and blue markers highlight wet periods (drought.gov). Vertical dashed lines denote temporal regime shifts

Like WCAs 3A and 3B, groundwater elevations in the ENP were also characterized by regime shifts in the years 2006 and 2016 (Fig. 4). During the period 1997–2005, groundwater elevation showed strong directional coupling with P-ET with both metrics increasing concurrently. Groundwater and P-ET both display positive slopes, with cumulative residual groundwater accumulating up to 20 ft above baseline (1997–2005) while responding positively to favorable natural water conditions and complementary managed flows. The reduction phase (2006–2015) showed that groundwater declined steadily despite relatively stable and elevated P-ET conditions. This suggests diminished influence of natural inputs on groundwater dynamics during this period, with the system continuing to lose water despite not experiencing proportional negative natural water balance. Groundwater elevations decreased by up to 15% from 2006 to 2015 compared with those from 1997 to 2005, particularly in dry months (March–May) (Table 2).

The reversal phase (2016–2022) showed that groundwater elevations began recovering despite below-average P-ET compared to the long-term mean. During this period, average groundwater elevation in the ENP exceeded 1997–2005 levels by up to 27%. This reversal coincided with periodic increases in managed water release, particularly evident during the 2017 wet period onset and the 2020–2021 strong wet period. Droughts during the reduced phase produced temporary perturbations in the cumulative groundwater trend, but the system’s long-term trajectory appears increasingly influenced by water management decisions (correlation between cumulative residual groundwater elevation and managed water volume between 1997 and 2022, *r* = 0.86) than natural variation in P-ET alone (*r* = 0.18).

**Fig. 5:**
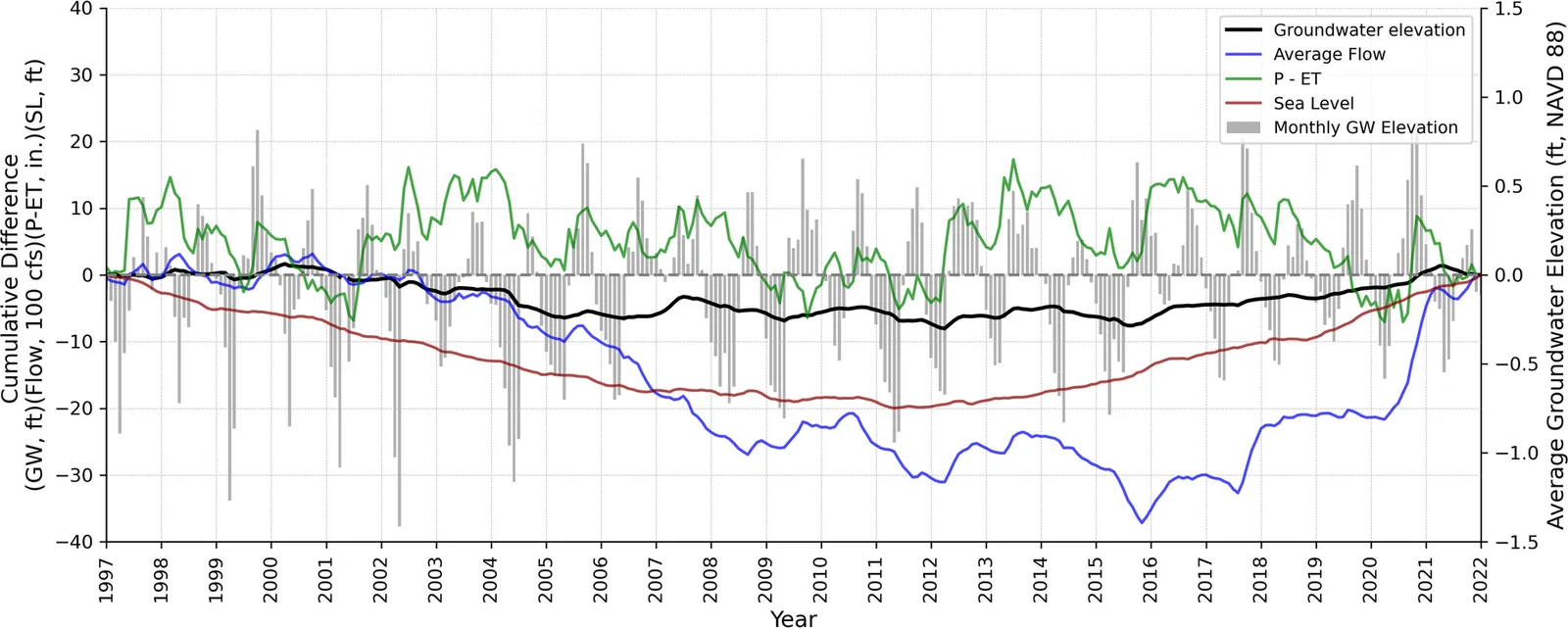
Residual mass curve of hydrological variables in Taylor Slough and C-111 coastal watershed (1997–2022). The primary *y*-axis shows cumulative differences from monthly means for average groundwater elevation (*n* = 5), average flow, precipitation minus evapotranspiration (P-ET), and sea level, while the secondary *y*-axis shows monthly average groundwater elevation (grey bars)

The lower portions of the ENP, including TS and C-111 coastal watersheds, were also largely influenced by managed water volume in coastal areas (*r* = 0.81) and cumulative residual sea level (*r* = 0.89) (Fig. 5). The degree of association with P-ET was low (*r* = −0.009).

Surface water stage trends (*n* = 36) in the ENP also resulted in statistically significant increasing trends in most stations (*n* = 7) located in Shark River Slough between the years 1997–2022 (average rate = +0.0438 ft/year.). In contrast, a decreasing trend in one station (NP–205) located downstream of S12A on L-29 was observed. Overall average surface water stage in the ENP was at its highest during 2016–2022, similar to groundwater elevation.

### Interannual and seasonal variability

#### Water Conservation Areas (WCAs) 3 A and 3B

Seasonal analysis revealed dampened variability in groundwater and surface water compared with rainfall. The peak rainfall occurred in June, whereas surface and groundwater elevations peaked in October. The wet season for rainfall in these watersheds (WCA 3A and 3B) spans May to October, whereas for surface water stage and groundwater elevation the high-water period can be delineated from July through January. Seasonal correlations were strong between groundwater and surface water (*r* = 0.99), with rainfall showing the strongest correlation at a 3-month lag (*r* = 0.89).

The residual groundwater elevation trends highlighted the influence of short-term hydrometeorological controls. Extreme weather events, including hurricanes and tropical storms, increased groundwater recharge. For example, Hurricane Irma (September 2017) and Tropical Storm Arlene (June 2005) occurred during periods of significant groundwater elevation increases (Figs. 3 and 4).

#### Everglades National Park

Seasonality in the ENP groundwater and surface water was similar to that in the upstream WCAs 3A and 3B, with the wet season for rainfall spanning May to October. For surface water stage and groundwater elevation, the high-water period occurred from mid-June to December. The seasonal correlations between surface water and groundwater were strong (*r* = 0.99), and the strongest seasonal correlation between rainfall and surface water/groundwater occurred with a 2-month lag (*r* = 0.87).

Three notable recharge years were identified, with the highest recharge observed in 2022 (11.27 cm/year), followed by 2021 (10.03 cm/year) and 2016 (8.56 cm/year). These years represent periods of substantial groundwater increase associated with above average rainfall, which may have contributed to increased P-ET, increased flow, or both. These results are in line with our understanding of the system where the surficial aquifer system is primarily recharged by rainfall and overlying surface water (Bloetscher et al., 2010; Price & Swart, 2006). Significant depletion periods were also observed, with the most severe depletion occurring in 2011 (−15.71 cm/year) and 2007 (−9.07 cm/year). These observed patterns align with well-known periods of hydrological extremes (droughts) in the region and emphasize the importance of continued monitoring and analysis to inform water resource management and continued restoration efforts.

### Spatial variability in groundwater elevation and surface water stage

#### Spatial interpolation of groundwater elevation

**Fig. 6:**
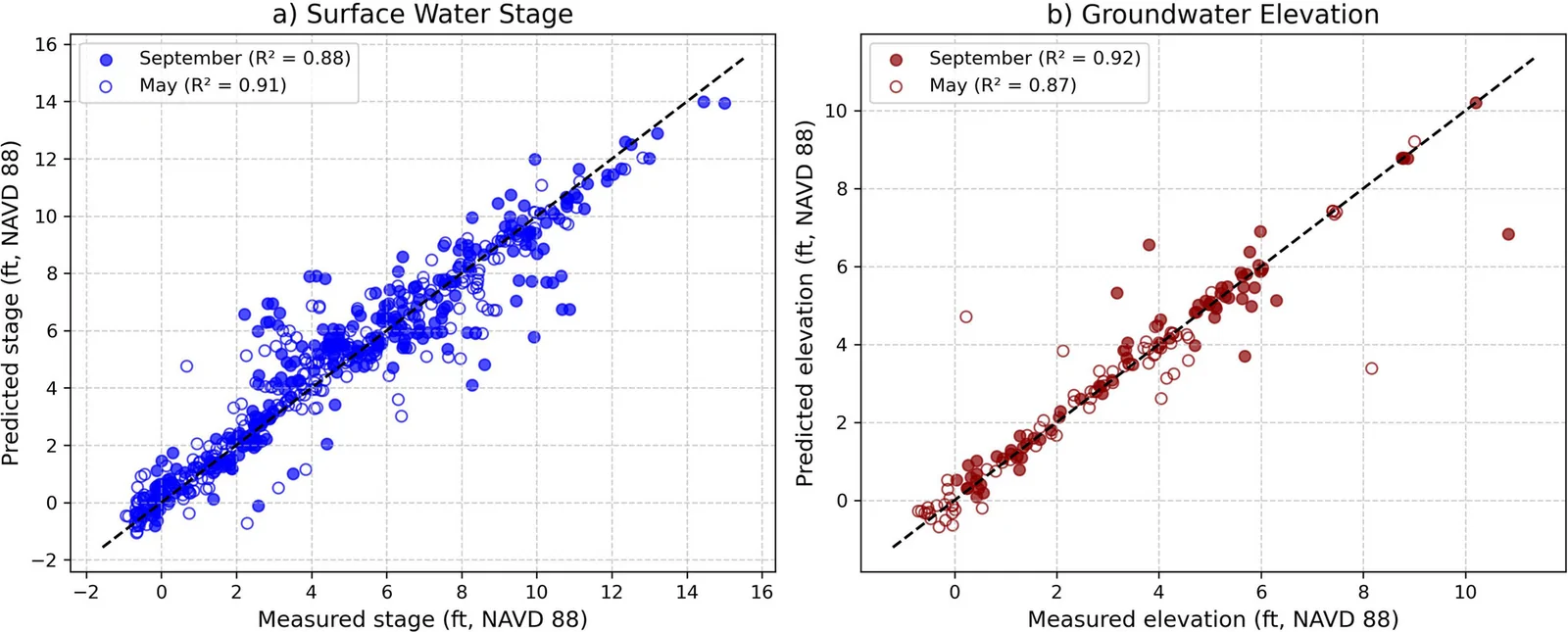
Long-term average (1997–2022) measured vs. predicted surface water stage (**a**) and groundwater elevation (**b**) via empirical Bayesian kriging (EBK) interpolation. September and May are shown

The spatial interpolation results of the monthly average (1997–2022) groundwater elevation for a typical wet month (September) and dry month (May) are shown in Fig. 6. The long-term (1997–2022) interpolated groundwater elevation during the wet and dry months resulted in RMSEs of 0.841 and 0.715, respectively. For the long-term average dry month and wet month, approximately 90% and 89% of the predictions were within the 90% prediction interval, and 91% and 96% of the predictions fell within the 95% interval, respectively. A considerable deviation from the observed groundwater elevation was noted in the East Collier watershed, where groundwater elevation was underestimated in C-296 and overestimated in C-995. These errors in estimation are due to the lack of groundwater elevation measuring stations in the surficial aquifer system in East Collier watershed, with the exception of stations C-296 and C-995. Uncertainties are also present in parts of the ENP where groundwater elevation wells are sparse.

**Fig. 7:**
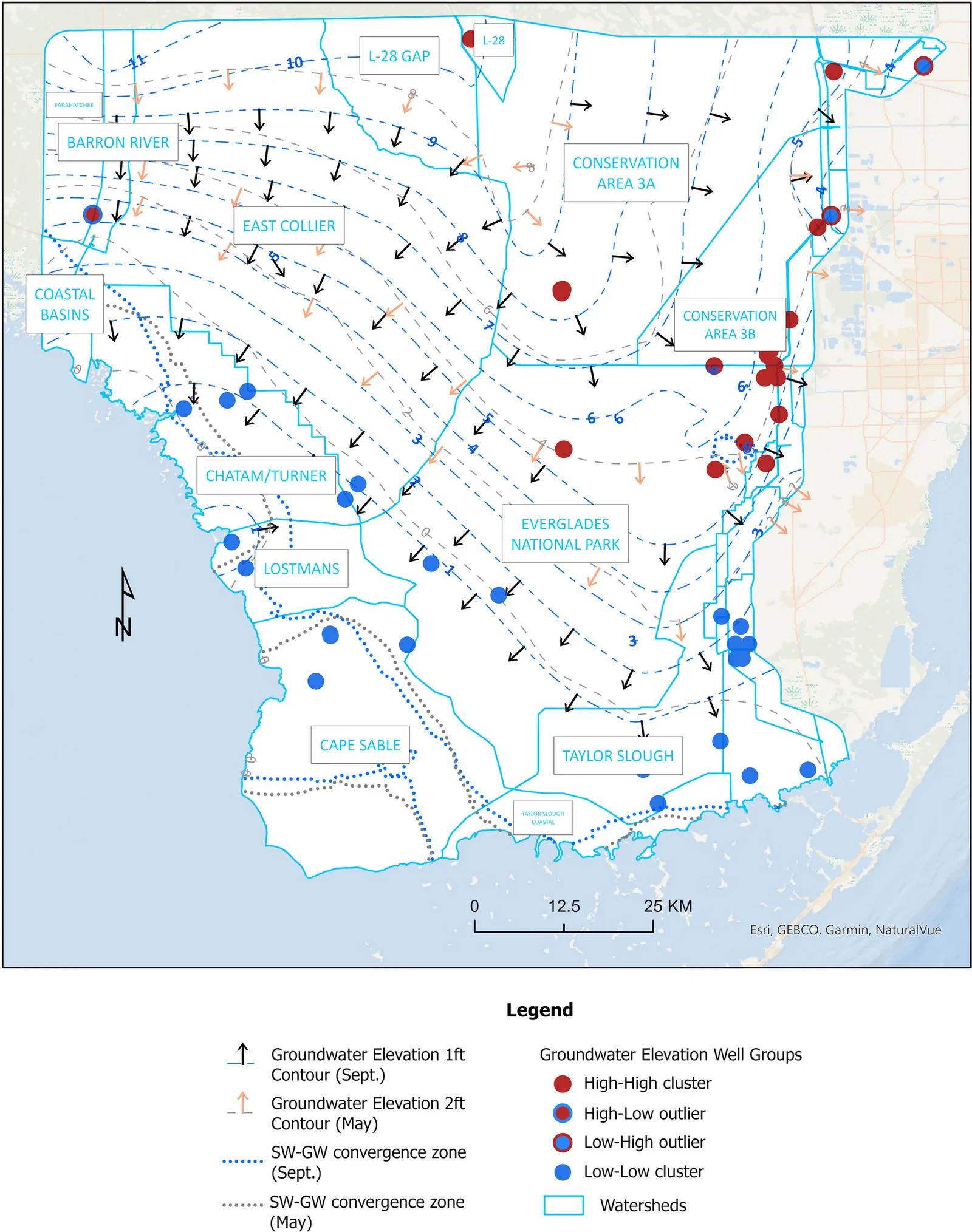
Long-term average (1997–2022) groundwater elevation patterns and flow directions across the study area. Groundwater elevation contours at 1-ft intervals for September (blue dashed lines) and May (orange dashed lines) represent wet and dry season conditions, respectively. Black arrows indicate groundwater flow directions determined from potentiometric gradients. Dotted lines delineate SW-GW convergence zones where surface water stage and groundwater elevation approach equivalence. Monitoring wells are classified by local indicator of spatial association: High-High clusters (well groups) (red circles), Low-Low clusters (blue circles), High-Low outliers (red with blue outline), and Low-High outliers (blue with red outline)

The interpolation revealed that higher groundwater elevations are consistently found in the northern part of the study area (East Collier, L-28 GAP, WCA-3A, and WCA-3B) across all months ranging up to 10.5 ft NAVD 88. In contrast, a gradual reduction in groundwater elevation is observed further downstream (Chatham Slough, Lostmans Slough, Cape Sable, TS, and the C-111 coastal watershed). The groundwater elevation during May reduced to below 0 ft NAVD 88 in these downstream watersheds, indicating a significant decrease during the dry season. The spatial configuration of groundwater potentiometric surfaces identified regional flow directions (Fig. 7). In East Collier and L-28 GAP (Central glades and Big Cypress), the hydraulic gradient indicates a southwestern flow direction with values ranging from 1.62 to 1.72 × 10^−4^ ft/ft during the dry and wet season, respectively. Within WCA-3A and 3B, groundwater contours have an eastward flow along the eastern boundary, with hydraulic gradients of 1.21–1.23 × 10^−4^ ft/ft.

Further south in the ENP region, groundwater flow patterns move south to Cape Sable and Lostmans Slough while a south-southeasterly flow develops towards TS where hydraulic gradients range from 1.18 to 1.24 × 10^−4^ ft/ft. These hydraulic gradients, all within an order of magnitude of 0.0001 ft/ft, reflect the characteristically flat topography of the region while maintaining consistent flow direction patterns across seasonal changes.

The SW-GW convergence zones, delineated in dotted lines (Fig. 7), designate areas where surface water stage and groundwater elevation approach equivalence. These convergence zones extend more inland in September (wet season) compared to May, particularly in TS.

The spatial autocorrelation of long-term average (1997–2022) groundwater elevation consistently revealed strong positive spatial autocorrelation across all months, with Moran’s I values ranging from 0.78 to 0.83 (*p* < 0.001, Table 3). These high values indicate that groundwater elevations are spatially clustered, meaning that wells with similar groundwater elevations tend to be geographically close to one another. The expected index (−0.01) and consistently large positive z scores (ranging from 13.18 to 14.07) confirm that the observed spatial patterns are highly unlikely to have occurred by chance.

**Table 3:** Spatial autocorrelation test for groundwater elevation using long-term average (1997–2022) groundwater levels (*p* value < 0.001)

	**Jan**	**Feb**	**Mar**	**Apr**	**May**	**Jun**	**Jul**	**Aug**	**Sep**	**Oct**	**Nov**	**Dec**
Moran’s Index	0.80	0.83	0.83	0.82	0.81	0.8	0.78	0.79	0.80	0.80	0.81	0.82
z score	13.65	14.06	14.07	14.02	13.77	13.18	13.29	13.40	13.59	13.61	13.80	13.89

The slight monthly variations in Moran’s I values suggest seasonal influences on the spatial distribution of groundwater elevation. For example, marginally lower values in the peak season (June–August) could reflect increased hydrological connectivity and recharge, which may reduce spatial heterogeneity. Conversely, higher values during the dry season (months November–April) may indicate stronger spatial clustering due to reduced recharge and more localized influences on groundwater elevation.

The analysis of local indicators of spatial association identified specific groundwater monitoring wells as spatial outliers. Well G-1637 showed Low-High (LH) patterns during both September and May, indicating locations where groundwater elevations were lower than surrounding wells with higher elevations. Wells C-995 (September) and C-296 (May) showed High-Low (HL) patterns, representing points with higher groundwater elevations than their surrounding neighbors with lower values.

The well group analysis revealed distinct spatial patterns in groundwater elevation (Fig. 7). High-High (HH) well groups were predominantly located along the eastern boundary of the study area, identifying regions where wells with above-average groundwater elevations are spatially concentrated. These well groups were primarily found in WCA-3B and adjacent areas. Conversely, Low-Low (LL) well groups were concentrated in the western and southern coastal regions, including Lostmans Slough, Cape Sable, and Taylor Slough watersheds, indicating areas where groundwater elevations are closer to or below the NAVD 88 datum.

**Fig. 8:**
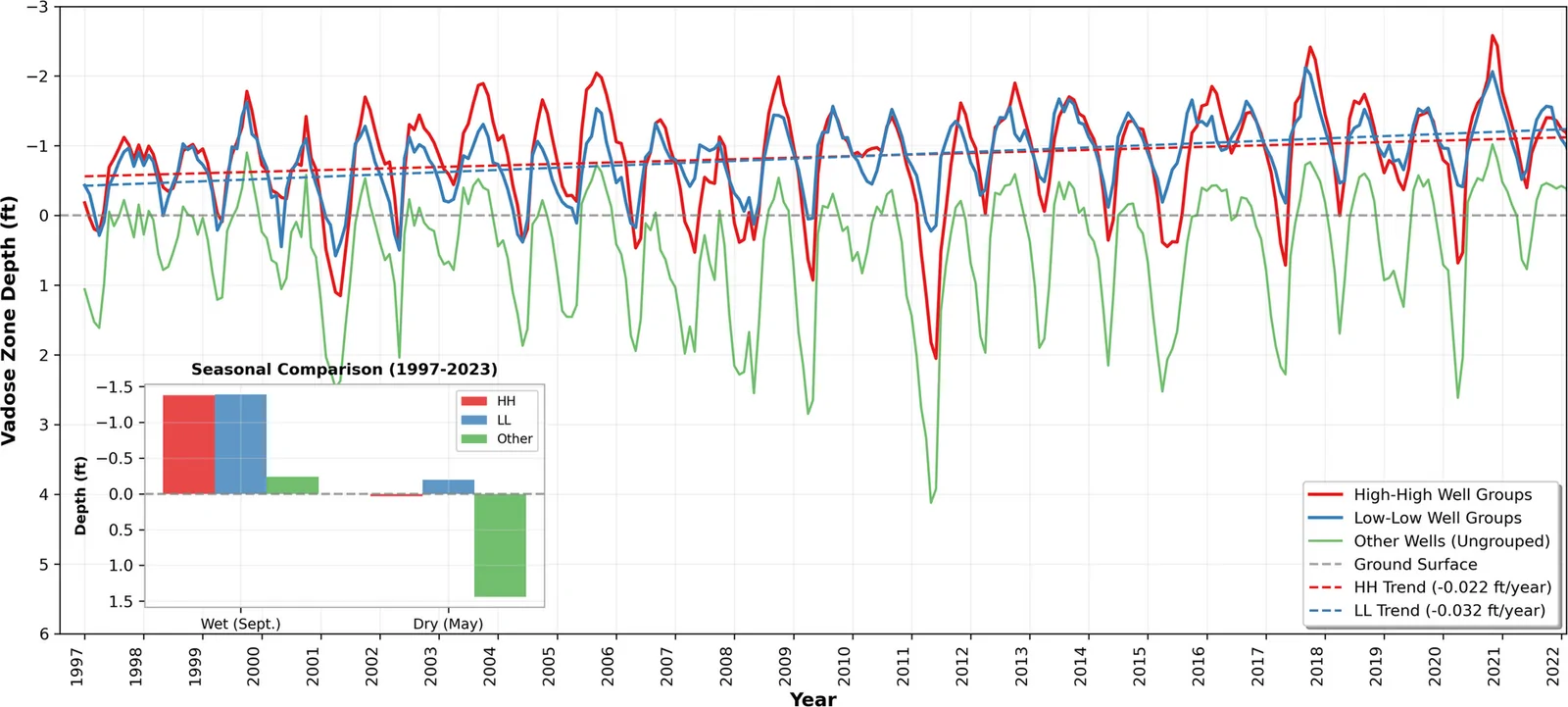
Temporal and seasonal patterns of unsaturated depth across well groups (1997–2022). Monthly average vadose zone depth for High-High well groups (red), Low-Low well groups (blue), and ungrouped wells (green). Positive values represent water levels below ground surface (unsaturated conditions). The dashed grey line represents the ground surface. The inset bar compares average conditions during the wet season (September) and the dry season (May) to show consistent inundation versus seasonal fluctuation

Examination of saturation conditions revealed that despite their classification as LL well groups based on elevation values, the coastal wells in Lostmans, Cape Sable, and Taylor Slough watersheds remained fully saturated throughout the year with minimal vadose zones (Fig. 8). This indicates that the LL designation reflects relative elevation differences rather than unsaturated conditions.

#### Spatial patterns and relationships between surface water stage and groundwater elevation

**Fig. 9:**
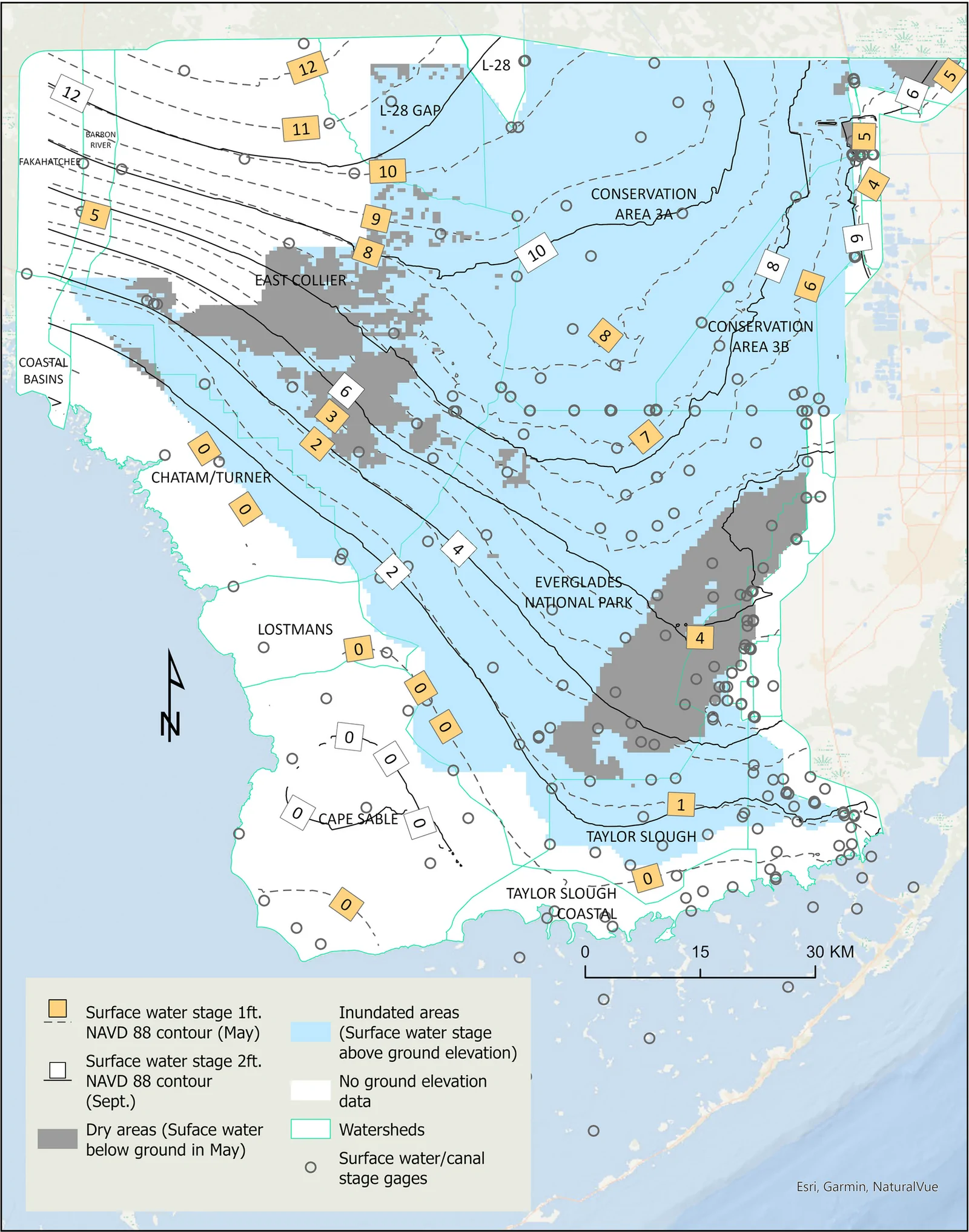
Long-term average (1997–2022) surface water stage patterns across the study area. Surface water stage contours at 2-ft intervals for September (solid) and 1-ft for May (dashed) represent wet and dry season conditions, respectively. Grey areas indicate regions where surface water falls below ground elevation in May, while light blue areas remain inundated. Circles indicate surface water stage monitoring gages used in spatial interpolation

Spatial analysis of surface water stage showed a north-to-south gradient ranging from 12 ft NAVD 88 in the WCAs 3A and 3B to near 0 ft in coastal regions (Fig. 9). Seasonal comparison showed surface water stage reductions during May compared to September, with western areas in East Collier watershed and portions of the ENP experiencing complete drying (surface water below ground elevation). The central flow path maintained inundation year-round. Notable regional differences included consistently low stages near 0 ft NAVD 88 in Cape Sable and Lostmans Slough, contrasting with the slightly elevated Taylor Slough (close to 1 ft NAVD). These patterns align with groundwater elevation contours (Fig. 6), particularly in WCAs 3A and 3B where both surface and groundwater follow similar hydraulic gradients.

The similarity in spatial patterns between surface water stage and groundwater elevation also implies a high likelihood of correlation. Pearson’s correlation analysis between monitoring stations substantiates this conjecture, showing a notable correlation between most surface water stages and groundwater elevations for the study period (1997–2022). Strong correlations (*r* ≥ 0.8) between surface water stages and groundwater elevations in the WCA 3A and 3B were observed at 92% of the monitoring stations, with values ranging from 0.77 to 1.00. The consistently high correlation among monitoring stations in WCAs 3 A and 3B indicates high similarity in the timing and occurrence of changes in the surface water stage. For example, the influence of the water level in canal C-304 measured at control structures S-151 and S-340 showed a very high correlation (*r* = 0.86–0.97) with G-968 and wells within WCA-3B. A relatively reduced correlation (0.7–0.8) is observed between monitoring stations spaced at relatively large distances (e.g., 39 KM between G-968 and S344 resulted in *r* = 0.63).

Among the evaluated correlations between the surface/canal water stage and groundwater elevations in the ENP, about 62% had a Pearson’s correlation coefficient (> 0.8). Canal stage elevation was also strongly correlated with groundwater elevation, with 60% of the variable pairs showing correlations above 0.8 and 34% falling between 0.6 and 0.8. These correlations indicate that groundwater elevation is strongly associated with the surface water stage in the ENP, with canal seepage potentially contributing to aquifer recharge and increased groundwater elevation in nearby wells.

These spatial patterns during both dry and wet months were further evaluated via a localized relationship. The analysis revealed three predominant interaction patterns between the surface water stage and groundwater elevation.

**Fig. 10:**
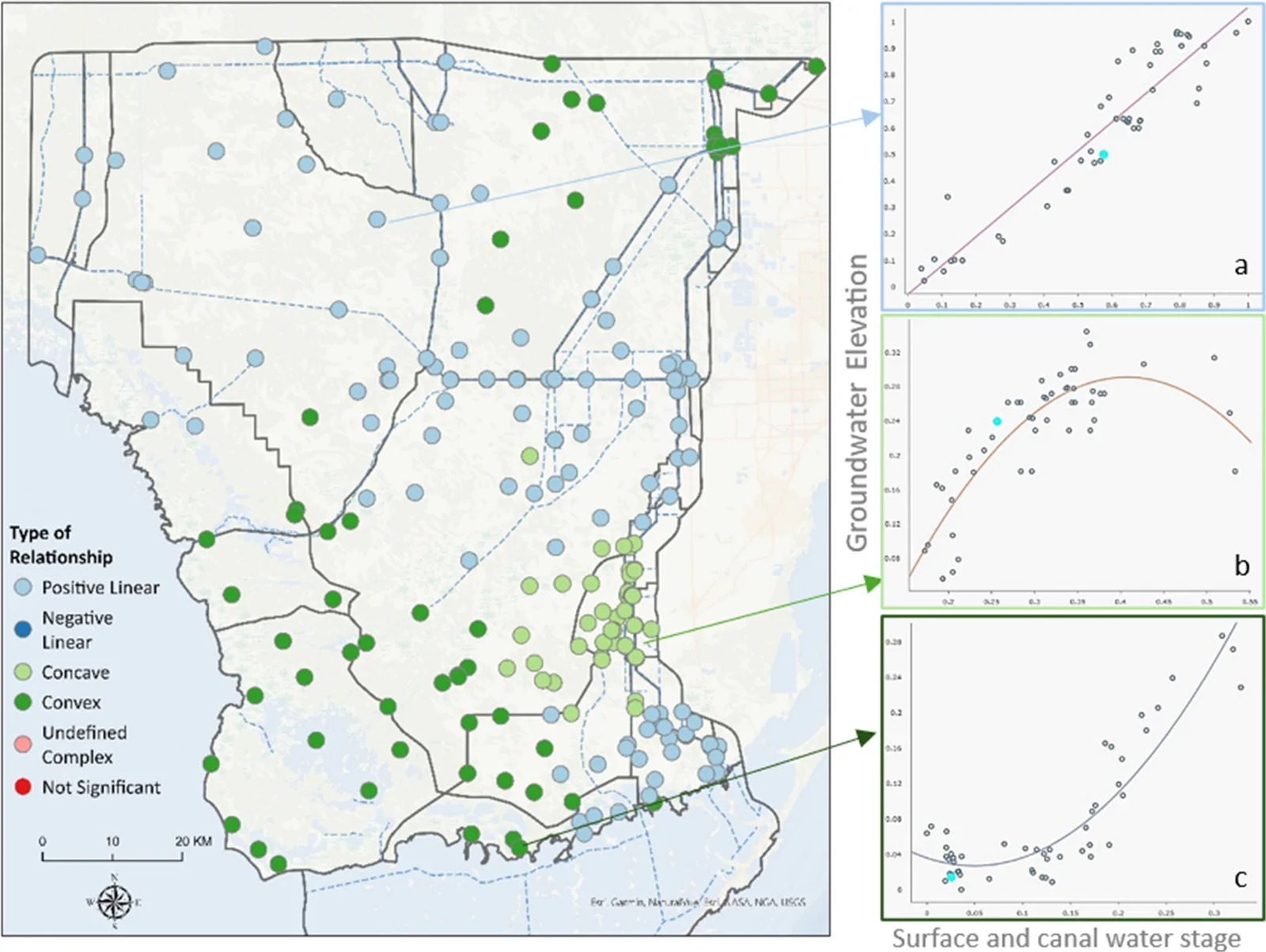
Spatial variability in the relationship between long-term average (1997–2022) surface water stage and groundwater elevation in the study area. A conceptualization of the relationships is shown on the right with **a** linear, **b** concave, and **c** convex polynomials. Surface/canal water stage gages are used to display the type of relationship on the map with interpolated groundwater elevations extracted over these points

Positive linear relationships (Fig. 10a): This was the most dominant pattern, covering 53.6% of the study area in May and 60.6% in September. Strong linear relationships (*R*^2^ > 0.85) were prevalent in East Collier and the northern portions of the ENP. Weaker fits were observed in parts of the TS coastal watershed, indicating the presence of heterogeneity in linear surface-groundwater connectivity across the region.

Convex relationship (Fig. 10c): Convex interactions, characterized by a steeper rate of change in groundwater elevation at higher surface water stages, were the second most common pattern. These were predominantly found in coastal watersheds, including Lostmans, Cape Sable, the lower ENP, and portions of the TS watershed. The strength of the convex relationships varied seasonally, with higher polynomial *R*^2^ values (0.88–0.97) observed during the wet period in Cape Sable, Lostmans, and the lower ENP. During the dry period, the *R*^2^ values decreased in coastal zones but improved in WCA-3A.

Concave relationship (Fig. 10b): Concave relationships observed in approximately 20% of the study area were defined by a rapid increase in groundwater elevation with increasing surface water stage, followed by a decreasing rate of change as groundwater levels approached saturation beyond a certain threshold. These patterns were localized to southeastern ENP (polynomial *R*^2^: 0.7–0.8) and sections of the upper TS near the L-31W canal. This pattern suggests that in these areas, groundwater elevation increases with the surface water stage up to some threshold, beyond which further increases in the surface water stage do not result in significant changes in groundwater elevation.

#### Temporal trends and seasonal variations in well groups

The groundwater monitoring wells utilized in this study were subsequently divided into two representative groups: High-High (HH) well groups and Low-Low (LL) well groups. The time series decomposition discussed in the previous sections was applied to each group of clusters to consider the spatial context and identify differences, if any, over time. The corresponding unsaturated depths in these areas were also evaluated (Table 4).

**Table 4:** Long-term average groundwater elevation and seasonal variability in the HH and LL wells

Metric	HH wells	LL wells
Overall trend		
Mean trend GW elevation*	5.83	0.26
Slope (change in ft per year)*	0.017436***	0.017484***
Total change in GW elevation (1997–2022)	0.27	0.29
Seasonality		
Maximum seasonal GW elevation*	0.65	0.5
Minimum seasonal GW elevation*	−0.81	−0.49
Range (variability)*	1.46	0.99
Seasonal peak month	October	September
Average unsaturated zone		
Mean depth of unsaturated zone (1997–2022) (ft)	−0.56	−0.61
Standard deviation (1997–2022) (ft)	0.78	0.60
Maximum seasonal depth to water table (ft)	0.24	0
Minimum seasonal depth to water table	0	0

***Statistically significant (*p* value < 0.001)

*Units represent the decomposed time series components (ft, NAVD 88) and not absolute values; therefore, they should be interpreted as a function of the raw time series

The wells identified as HH include 3AS3-GW4, G-620_B, G-968, G-3576, S356GW3, 3AS3-PZ2, 3BS1-GW2, 3BS1-GW4, G-3272, 3BS1-PZ4, C-54, G-618_B, 3BS1-PZ3, 3AS3-GW2, G-2032, L31NNGW4, 356GW1, G-1488, 3AS3-PZ3, and G-3574. These wells are situated upstream in the northern parts of the ENP and WCAs 3 A and 3B and are influenced primarily by freshwater recharge and upland hydrology. In contrast, LL wells located downstream in lower-lying coastal areas include G-3353, G-3777, G-3764, MO-214, ENPLHC, MO-216, MO-211, ENPLJB, CH1, CH2, CH3, LO1, LO2, LO3, LO4, SH1, SH2, SH3, SH4, and SH5, which are subject to the combined effects of sea level rise (SLR), upstream flows, and rainfall.

The analysis of groundwater elevation trends revealed a greater mean elevation in the HH wells (5.83 ft, NAVD 88) than in the LL wells (0.26 ft). This may reflect the generally greater topographical position of HH wells and their likely greater interaction with surface water recharge in upland regions. Further analysis of recharge rates would clarify these differences. Despite similar rates of elevation change over time (0.017 ft/year for both the HH and LL wells), the total increase in groundwater elevation between 1997 and 2022 was slightly greater for the LL wells (0.29 ft vs. 0.27 ft). The rise in HH wells is likely due to enhanced recharge from rainfall or upstream hydrological connectivity, whereas in LL wells, SLR and a reduced vadose zone likely play a more prominent role.

Seasonal analysis showed minimal overall differences in magnitude, suggesting that seasonal fluctuations are minor compared with the overall trend. The seasonal variation in groundwater elevation differed moderately between the two groups of clusters. Compared with the LL wells (0.99 ft), the HH wells presented a broader seasonal range (1.46 ft), indicating a more dynamic hydrological response driven by wet-season recharge and surface water inputs. Seasonal peaks in HH wells occurred in October, aligning with the late wet-season dynamics. In contrast, LL wells peaked in September, potentially indicating earlier hydrological responses linked to tidal/coastal influences. The delayed peak in HH wells might suggest prolonged recharge dynamics and vadose zone buffering upstream. The HH clusters are generally expected to contribute to the flow toward the LL wells. However, the fact that HH wells peak later in the year (July–January, with an October peak) than LL wells do (which peak from June–December, with a September peak) indicates that the flow dynamics might be influenced by factors other than mere upstream to downstream transfer.

In examining the reasons behind these unexpected seasonal peak timings, further analysis suggests that local factors likely play a greater role. For example, in areas such as Chatham and Lostmans Sloughs, the September peak aligns with high rainfall during that period, as these regions receive greater rainfall than other regions in the study area. Additionally, the seasonal sea level trends at Naples, with peaks occurring between July and September, further support the potential association with sea level fluctuations at these locations. LL wells near coastal zones are also sensitive to tidal influences (e.g., September king tides). Tidal peaks and localized rainfall effects could combine to produce earlier seasonal maxima.

The average depth of the unsaturated zone in both the HH and LL areas indicated overall saturated conditions. Notably, the maximum seasonal depth to the water table reached 0.24 ft in the HH areas, whereas the LL areas were consistently saturated throughout the year. The prolonged inundation in LL areas is likely due to low elevation, SLR, and tidal effects. These conditions are also present during dry years. Short-term deviations from the saturated depth were apparent in HH areas during 2009, 2011, and 2020, whereas LL areas remained saturated during these periods and exhibited less variability.

The geometric mean of hydraulic conductivity for LL areas in the Shark River Slough and TS coastal watersheds, as reported in Cordoba (2022) and Harvey (2000), is 60 ft/day and 1.1 ft/day, respectively. In HH areas, it is approximately 30,000 ft/day (Fish & Stewart, 1991). These stark differences in aquifer properties also corroborate the more immediate response from HH wells during recharge or reversal periods. The relatively short duration of the response to depletion events suggests resilience within the system, as evidenced by a subsequent recovery in groundwater elevation (Figs. 3 and 4). This recovery is likely driven by a combination of natural aquifer recharge processes and potential water release following termination of drought-induced reductions in groundwater elevation.

These results reveal the distinct regional hydrological dynamics within the surficial Aquifer System. In HH wells, the seasonal variability and greater vadose zone buffering capacity suggest that upstream freshwater recharge plays a role in maintaining groundwater levels. In LL wells, with their narrower seasonal range and consistently saturated conditions, the increasing influence of SLR on coastal aquifer dynamics is more likely.

### Surface water stage variability in FL Bay

The analysis of the degree of association between Florida Bay water levels and potential driving factors revealed several patterns. Pearson’s correlation analysis showed consistently positive correlations (0.82–0.91) between canal water stage and Florida Bay surface water stage, particularly downstream of S197 and S18C. These correlations remained high during the dry season (0.78–0.94) but diminished slightly (0.56–0.61) at upstream locations within the canal (S177). Groundwater elevation in TS and C-111 subwatersheds also demonstrated significant correlation with Florida Bay surface water stage, particularly at monitoring stations near the bay.

**Fig. 11:**
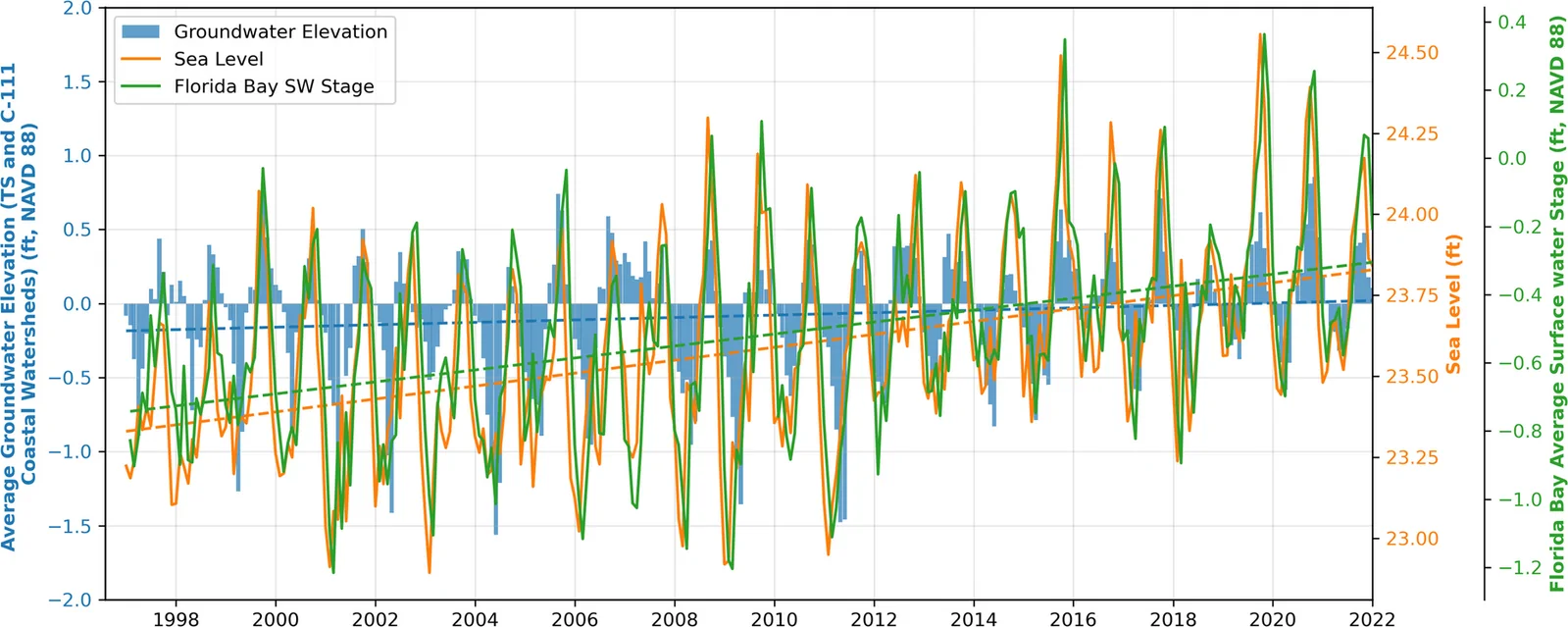
Monthly time series (1997–2022) of groundwater elevation in Taylor Slough and C-111 coastal watersheds, sea level, and Florida Bay surface water stage. Dashed lies represent linear trends indicating long-term increases in all components

Long-term average monthly surface water stage in Florida Bay derived from stations ENPDK, ENPWB, ENPBN, ENPPK, ENPBA, ENPLR, ENPBK, MBTS, ENPJK, ENPLB, and ENPBS (Fig. 2) ranged from −0.6 ft in the dry period to 0.5 ft NAVD88 in the wet period. The long-term average monthly surface water stage in Florida Bay resulted in a progressive increase in water levels over time across all months (Fig. 11), with a significant increasing trend (*p* value = 0) in the water level in Florida Bay, with a slope of 0.0017 ft/month.

Surface water stage patterns in Florida Bay differed from upstream areas (ENP and WCAs 3A and 3B) in two ways. First, while upstream areas consistently showed lowest elevations in May and peaks in October across all periods (1997–2005, 2006–2015, 2016–2022), Florida Bay peaked in September during 1997–2015 but shifted to October in 2016–2022, with lowest stages occurring from January to March. Second, the groundwater elevation and surface water stage in upstream areas are generally aligned, with increases in the surface water stage reflected in corresponding groundwater elevation increases. However, in Florida Bay, the surface water stage did not consistently mirror changes in discharge from coastal creeks. Compared with other periods, coastal creek discharge, which we consider to account for the variability in both surface water stages in coastal watersheds and flows from upstream areas, was generally highest from 1997 to 2005 during the wet period (June–October). Discharge decreased by up to 80 cfs. on average between 2006 and 2015 and increased by up to 40 cfs. during the period 2016–2022. Between 2016 and 2022, average discharge from coastal creeks surpassed the original 1997–2005 levels during the dry season (November–May).

#### Drivers of water level variability in Florida Bay

Analysis of the random forest regression model showed key factors influencing surface water stage variability in Florida Bay from 2000 to 2022. 

The model showed strong performance with consistent RMSE values across all four cross-validation folds (Fold 1—RMSE: 0.087; Fold 2—RMSE: 0.094; Fold 3—RMSE: 0.088; Fold 4—RMSE: 0.064; and average cross-validation RMSE: 0.083). These results suggest that the model is effective at capturing the temporal dynamics of surface water stage changes, with low errors across different segments of the training data. The low RMSE values are particularly important because they indicate that the model is reliable even in the presence of temporal variability in the data. 

RMSE for the test period (2017–2022) was 0.074, and *R*^2^ = 0.896, which is consistent with the cross-validation results and is considered adequate for the analysis. Diagnostic tests confirmed that model residuals satisfied all statistical assumptions: normality (Shapiro-Wilk test: *p* = 0.4076), independence (Durbin-Watson statistic = 2.24), homoscedasticity (Breusch-Pagan test: *p* = 0.3309), and stationarity (augmented Dickey-Fuller test: *p* < 0.001). 

The feature importance analysis revealed that sea level was the most important driver of the surface water stage in Florida Bay, accounting for 88% of the total importance in the model. The remaining drivers contributed a combined 12% of the total feature importance in the model (Table 5).

**Table 5:** Relative importance of drivers in surface stage level changes in Florida Bay

Driver	Relative Importance
Sea level	88.30%
Wind speed	3.12%
Rainfall	2.59%
Groundwater elevation	2.39%
Creek discharge	2.19%
Evaporation	1.40%

## Discussion

The analysis of spatiotemporal patterns in groundwater elevations within South Florida, particularly in WCAs 3A & 3B and ENP, provided insights into the hydrological dynamics of these ecologically sensitive regions. The findings show the influence of both natural processes and water management practices on groundwater elevation, highlighting key trends, seasonal variability, and interconnectedness between groundwater and surface water. Ecological implications of these insights can inform management and restoration of the Greater Everglades. A primary goal of the Comprehensive Everglades Restoration Plan (CERP), the largest wetland restoration project in the world, is to restore ecosystem functionality by increasing upstream flows from the systemwide drainage that highly degraded the system.

### Trends in groundwater elevation

Long-term trends provide insights into the direction of groundwater elevation over extended periods. We identified clear shifts in groundwater elevation across years and seasons. The period 2006–2015 was identified as a reduction phase characterized by groundwater elevation and surface water stages falling below the long-term average (Figs. 3 and 4 and Table 2). In WCAs 3A and 3B, this phase included short, yet impactful depletion years (2007, 2009, 2011, 2015) followed by stable conditions (years 2016 and 2020). The period of 2016–2022 was identified as a reversal phase where groundwater elevations in all months increased (16%) beyond their initial 1997–2005 levels (Table 2).

Seasonal analysis revealed attenuation of groundwater and surface water patterns compared with rainfall. The surface water stage and groundwater elevation had similar seasonal magnitudes (quantity) and variabilities. The groundwater elevation peaks lag behind the rainfall peaks, reflecting the delayed response of the aquifer system to surface inputs. In WCAs 3A and 3B, the highest seasonal correlation of rainfall with groundwater elevation occurred at a 3-month lag (*r* = 0.89), whereas in the ENP, the strongest correlation occurred at a 2-month lag (*r* = 0.87), consistent with lagged correlations in Shark River Slough reported by Saha et al. (2012). This lag is critical for understanding water storage dynamics and availability.

Residual analysis revealed periods of below-normal groundwater elevations in most upstream areas (WCAs 3A and 3B and the ENP). These events lasted up to 15 months during 2006/2007 and 2000/01 and up to 13 months during 2010/11 and 2014/15, which aligned with drought years (drought.gov). Previous studies have shown that drought periods significantly affect groundwater drawdown and flow patterns, influencing resource availability and potentially compromising the effectiveness of restoration efforts in these regions (Harvey et al., 2004a, 2004b; Merritt, 1997; Nungesser et al., 2015; ICCG, 2009). Adequate hydroperiods are needed to support ridge and slough topography and tree island ecology as well as maintain peat soils in these areas (McVoy et al., 2011; Ogden, 2005). The groundwater elevation also exhibited responses to extreme weather events, such as 2012 (15 months, wet period), 2020/21 (12 months, after TS Eta in late 2020), 2017 (10 months, after hurricane Irma and Michael and Gordon), and 2005 (9 months, after three hurricanes). These events reflected rapid recharge, emphasizing their importance in recharging aquifers, particularly in areas where the surficial aquifer system is sensitive to rapid water influx. Downstream regions such as the Chatham, TS, Lostmans, and Cape Sable areas presented subdued responses to these events, likely due to lower hydraulic conductivity. For management, this indicates that recovery from declines is slower and weaker downstream, so the buffering provided by episodic recharge is unevenly distributed across the system. Coupled with low water elevations in the dry season, reduced hydroperiods can limit prey production supporting wading birds in these downstream areas that can take several years to recover (Herring et al., 2011; Trexler et al., 2005). While extreme periods provide episodic recharge, offsetting long-term declines, reliance on such events for recharge may not be sustainable.

### Recharge dynamics in upstream freshwater marshes

The observed spatial relationship between surface water stages and groundwater elevations in WCAs 3A and 3B highlights the responsiveness of groundwater to substantial surface water increases, especially during the dry period. This connection is critical; however, temporal trends show only modest increases during 2016–2022 in surface water stage in WCAs 3A and 3B, while groundwater elevations have risen beyond their 1997–2005 levels. This increase in groundwater elevation, despite declining rainfall, could, in theory, be driven by increased lateral subsurface inflows, enhanced infiltration, increased recharge practices, and increased seepage. A much higher degree of association between surface water and groundwater levels with flows compared to natural factors (P-ET) highlights connection between the two.

Changes in groundwater levels were most pronounced in ENP, where groundwater levels had a significant increasing trend after 2016. During the reduction period (2006–2015), groundwater levels decreased by up to 15% compared with 1997–2005 levels, particularly during dry months. The recovery post-2016 exceeded prior levels, increasing to 27%. These changes align with the periodic shifts in average managed discharge, which decreased from 253 cfs. (1997–2005) to 155 cfs. (2006–2015) and rebounded to 259 cfs. after 2016. The role of inflow has also been reported to have driven over 55% of the variability in water level in upstream freshwater marshes in past studies (Dessu et al., 2018). The post-2016 groundwater recovery in ENP coincided with the increase in managed discharge, indicating that managed delivers are an effective means of raising groundwater levels in the area. Significant recharge years, including 2022, 2021, and 2016, contributed to the overall increase in groundwater elevation during this subperiod. Sustained higher water levels in these freshwater marshes may support wetland vegetation and dry-season habitat and potentially slow peat loss. The absence of significant depletion events in recent years suggests a potential shift in the groundwater balance and highlights the importance of continued monitoring to evaluate long-term sustainability in response to climatic and anthropogenic influences.

The high Pearson’s correlation between groundwater elevation and surface water stage within both WCAs 3A and 3B and ENP indicates a strong association between these hydrological components. The strong linear relationship between the surface water stage and groundwater elevation in upstream ENP regions further reflects this hydrological connectivity. Additionally, associations with overarching drivers and their potential cumulative effects were also observed. Temporal patterns in groundwater elevation, particularly recharge periods, were associated with P-ET and, to a greater extent, flow. The cumulative change in discharge compared with the long-term average followed a similar trend to that of groundwater elevation, suggesting an aggregated effect over time. In contrast, the association with cumulative P-ET (an estimate based on potential evapotranspiration) was weaker. Additional factors, such as seepage from levees and canals, may be associated with and contribute to aquifer recharge and thus considered when managing water resources in these areas.

### Spatial patterns of GW elevation

Spatial interpolation of groundwater elevation and surface water stages revealed consistent patterns across wet and dry months, with higher groundwater elevations in East Collier and WCAs 3A and 3B and a reduction downstream, which was especially pronounced during dry periods. The spatial distribution between groundwater elevation and surface water stage provides a comprehensive understanding of the hydrological interactions between surface-groundwater systems. The spatial interpolation of the monthly average groundwater elevation from 1997 to 2022 suggests that higher groundwater elevations throughout the year are consistently apparent in the East Collier, L-28 GAP, and WCA-3A regions. The spatiotemporal coherence observed in groundwater elevations and surface water stages across the study area is influenced by a combination of natural drivers, such as rainfall and hydrologic connectivity, as well as anthropogenic factors, such as canal operation and groundwater extraction.

Higher groundwater elevations in upstream areas may be attributed to greater rainfall inputs and hydrologic recharge in the region. Conversely, surface water dynamics, particularly canal systems such as the L-29 borrow canal, have a greater influence on groundwater elevation downstream in lower parts of the ENP. Canal operation, including water diversion and release, can directly affect groundwater recharge rates and flow patterns, especially in regions where surface water and groundwater systems are interconnected. This finding is necessary for understanding the hydrogeology and overall water balance within this region. This correlation implies that any changes in surface water elevation, whether due to climatic events or anthropogenic activities, are likely to have a proportional effect on groundwater elevation. The correlations also imply that managed water flows rather than rainfall or evapotranspiration primarily influence groundwater elevation, further indicating that targeted spatial and temporal adjustments to management flows could be used to support recovery from drought-driven declines identified here and more broadly, CERP.

Stable regional groundwater flow dynamics are shaped by topography and hydrological gradients. The minimal variability in these gradients indicates that the surficial aquifer system maintains its flow direction despite seasonal changes. Slight increases in the hydraulic gradient during the wet season suggest that protecting recharge zones could increase downstream groundwater movement under future management strategies. Proximity to canals and levees can artificially increase hydraulic gradients and influence groundwater flow directions (Harvey et al., 2000, 2004a, 2004b). Along the eastern boundary of ENP is the L31 canal, which separates ENP from the drier, human-inhabited areas to the east. Groundwater flow directions along the eastern boundary of ENP are typically toward the east or southeast away from ENP (Fig. 7). The long-term pattern of groundwater flow away from ENP along its eastern boundary is observed in both the wet and dry seasons (Fig. 7) compared to the historical flow pattern. These findings support multiple studies that have documented water flow directions away from ENP along its eastern boundary (Brown, 2015; Genereux & Slater, 1999; Kotun & Renshaw, 2014; Sullivan et al., 2014; Wilcox et al., 2004). However, increased water flows in the region seem to be having a positive influence on groundwater levels in ENP since 2016 despite a decrease in rainfall (Fig. 4).

### Upstream-coastal dynamics and diverging drivers of groundwater trends

Analysis of local indicators of spatial association identified statistically significant clusters of groundwater elevation, which were categorized into two groups: HH wells, which represent upstream freshwater marshes, and LL wells, which represent coastal areas. Our results highlight that coastal areas are fully saturated year-round, with negligible vadose zones. This finding shows that the designation of LL clusters reflects the relative groundwater elevation difference rather than the presence of unsaturated zones. The lack of vadose zones in these regions aligns with previous studies indicating the influence of sea level and saltwater intrusion on the coastal aquifer (Price et al., 2006). Furthermore, the reduction in unsaturated depth over time observed in both upstream and coastal regions suggests an increase in groundwater saturation.

Drivers behind these saturation trends appear to be spatially distinct. In upstream areas within freshwater-dominated regions of northern ENP, increasing saturation is likely linked to increased groundwater recharge via altered rainfall regimes or increased inputs of surface water flow. The groundwater elevation in these areas was more strongly associated with total flow (Fig. 4). Conversely, coastal regions show increasing saturation influenced by a combination of SLR, flow, and P-ET (Fig. 5). Groundwater elevation in these coastal areas is more strongly correlated with cumulative changes in sea level. This may have influenced the increased contribution of groundwater to surface water levels in coastal TS, which was noted by Sandoval et al. (2016). These findings support long-standing research on coastal aquifer vulnerability to SLR (Parkinson et al., 2015; Saha et al., 2011; F. H. Sklar et al., 2021; Xiao & Tang, 2019). A notable ecological impact has been mangrove encroachment from the southern coast northward (Ross et al., 2000). Areas along the eastern boundary are characterized by persistent unsaturated conditions and minimal seasonal variability likely driven by anthropogenic influences (with groundwater withdrawals averaging 5–65.8 MGM). These regions appear to be in hydrological isolation from broader groundwater fluctuations in the ENP, particularly from the context of saturation conditions. Contrasting upstream and coastal responses suggests that operational strategies would be most effective applied region by region. Controlled releases offer the greatest leverage over groundwater levels in the freshwater interior, whereas coastal zones may require factoring in sea level influence beyond local management.

### Hydrological connectivity and drivers of surface water stage variability in FL Bay

Observed spatial correspondence between surface water stages and groundwater elevation was supported by Pearson’s correlation analysis, which emphasized the interconnected nature of these hydrological components. However, the strength and nature of these relationships vary across the study area. Linear relationships dominate the northern ENP, reflecting strong surface-groundwater connectivity as documented by Harvey et al. (2004a, 2004b). In contrast, the convex and concave relationships observed in the coastal watershed and southeastern ENP indicate that groundwater response to surface water changes are not proportional across all water level ranges. These nonlinear patterns likely reflect localized thresholds where factors such as substrate permeability, topographic variations, or tidal influences create different rates of groundwater response at different surface water stages, highlighting the heterogeneity of surface-groundwater interactions influenced by geomorphological and hydrogeological factors.

The combined upstream influence (i.e., historical hydrologic patterns in WCAs 3A & 3B, ENP, and coastal watersheds) was used to understand the potential impact on the surface water stage in Florida Bay. Various factors, including freshwater inputs from sources such as rainfall, sheet flow, creek discharge, and potential groundwater flow, play a significant role in determining water level fluctuations within the bay (Stabenau & Kotun, 2012). Freshwater enters Florida Bay through channels such as TS and coastal creeks and periodically from discharge via S197 on canal C-111. The observed increase in coastal creek discharge during the dry season post-2016 may reflect restoration initiatives around 2016 that increased flows to the bay. These freshwater inputs are essential for sustaining the ecological balance of Florida Bay. Seasonal variation in water levels influenced by hydrological processes has significant implications for the ecology of Florida Bay. Fluctuations in water depth affect habitat and light availability, nutrient cycling, and species distributions (Boyer et al., 1999; Glibert et al., 2023). The relationship between freshwater discharge and water levels, which have broader implications for salinity levels, can vary across different regions of the bay because of factors such as topography and hydrological connectivity (Nuttle et al., 2000).

The increasing surface water stage trend in Florida Bay (1997–2022) suggests significant long-term hydrological changes. A shift in the peak water stage from September 1997–2015 to October 2016–2022 in Florida Bay may reflect altered hydrological inputs influenced by sea level peaks, consistent with seasonal sea level cycle of the adjacent Gulf of Mexico to which water levels in the bay are closely linked through substantial water mass exchange (Park et al., 2017).

Despite its role as a key conduit for freshwater inputs, creek discharge has shown limited explanatory power in influencing the surface water stage in Florida Bay. This finding suggests that while creek discharge contributes to the bay’s hydrological regime, its direct influence is foreshadowed by other drivers. Multivariate regression analysis identified sea level as the most critical driver of surface water stage variability in Florida Bay, accounting for 88.3% of the feature importance. This is consistent with other examinations in Florida Bay (Allen et al., in prep) and aligns with the established literature highlighting the role of sea level in influencing coastal dynamics (Chua & Xu, 2014; Dessu et al., 2018; Khojasteh et al., 2021).

The importance of sea level as the primary predictor aligns with its well-documented influence on estuarine and coastal environments, where sea level fluctuations directly impact water levels (Passeri et al., 2015). Previous studies have highlighted the connection between sea level variability in South Florida and global climate patterns, noting periods of rapid rise followed by more stable phases (Stabenau & Kotun, 2012). Sea level can act as a proxy for broader factors that impact the bay indirectly rather than serving as a direct flux into or out of the bay. Sea level can influence the water stage in coastal environments by increasing tidal inundation and flooding, which affect water flow dynamics. A rise in the water level can modify the exchange of water between the bay and the ocean, thereby influencing broader hydrological processes (Chua & Xu, 2014; Khojasteh et al., 2021).

Additionally, sea level can influence coastal groundwater elevation. Previous studies have highlighted the importance of diffuse groundwater seepage from surficial aquifers as a pathway for submarine groundwater discharge and a significant component of Florida Bay’s hydrological and nutrient balance (Swarzenski et al., 2009). Water-budget analysis of Shark River Slough also found significant net groundwater discharge into the slough, including a brackish, seawater-influenced component that is positively correlated with coastal surface water salinity (Saha et al., 2012). Elevated sea levels increase saltwater intrusion and contribute to shifting the hydraulic gradient between the bay and coastal systems, balancing or reversing the groundwater flow direction and volume (Swarzenski et al., 2009). Given this, the high importance of sea level in the model could reflect its role as an integrative proxy variable for multiple external influences.

Although the association of groundwater elevation in coastal watersheds with surface water stage in Florida Bay is strong, it contributes very little to changes in the surface water stage in the bay. Dominance of sea level over freshwater inputs in explaining surface water stage variability indicates that bay water levels are governed by climate-driven sea level trends within which managed freshwater deliveries are the operationally controllable input. These findings place local hydrologic changes within a broader environmental context. Projections of continued sea level rise represent an external influence on water level trajectory in the bay that lies beyond local management control and remains a central consideration for long-term outlook. Past studies have also indicated the vulnerability of the coastal portions of South Florida due to the increased frequency and magnitude of extreme sea level changes (Dessu et al., 2018; Obeysekera et al., 2011). Effective management requires a holistic approach that views sea level not only as a direct driver of surface water dynamics but also as a proxy for broader systemic pressures, such as tidal inundation, saltwater intrusion, and hydrological exchange, which collectively impact coastal environments. Furthermore, sea level serves as an integrative indicator of large-scale climate drivers, such as ENSO, which influences regional rainfall, storm activity, and drought cycles. Addressing these interconnected challenges demands strategies that bridge local management efforts with global climate adaptation frameworks.

## Conclusion

The hydrological dynamics of South Florida, particularly in WCAs 3A and 3B and ENP, reflect the interaction between natural processes and anthropogenic interventions. Historical modifications, such as canal construction and flood control measures, have profoundly altered groundwater and surface water interactions. Everglades Restoration is complex. Mitigating historical modifications for the benefit of Everglades Restoration requires an understanding of hydrological regime shifts, especially when biological cycles such as Apple Snail reproduction, or biogeochemical cycles, such as phosphorus accumulation due to soil oxidation, are driven by recurring hydroperiods and depths (F. H. Sklar et al., 2005). Long-term trends in groundwater elevation reveal statistically significant regime shifts, with distinct phases of accumulation, decline, and recovery driven by water management practices and climatic influences. The depletion phase (2006–2015) was characterized by groundwater level declines attributed to both reduced flow and prolonged dry periods, whereas the recovery phase (2016–2022) exceeded prior levels, indicating shifts in managed discharge and enhanced aquifer recharge. Seasonal analysis revealed strong correlations among rainfall and groundwater elevation with lag times, emphasizing that the response of the aquifer systems to surface water inputs was delayed. Short-term anomalies, including droughts and storms, contribute to fluctuations in groundwater elevation. Fluctuations in groundwater along with their associated lag times are expected to have a profound effect upon saltwater intrusions into the ENP and the transition of freshwater sawgrass marshes into saltwater mangrove swamps (F. H. Sklar et al., 2021).

Groundwater elevation in East Collier watershed, parts of WCAs 3A and 3B, and northern ENP showed strong linear relationship with surface water stages. Coastal areas and parts of the northern Taylor Slough and parts of WCAs 3A and 3B reflected nonlinear and localized nature of surface-groundwater interactions, which may be influenced by localized factors. Despite the decrease in rainfall, the groundwater elevation recovered in upstream freshwater marshes. It is not clear what has driven this trend. In theory, recovery could be driven by subsurface inflows, increased recharge practices, and increasing seepage. The greatest recovery in groundwater elevation was found in the northern ENP, potentially driven by increased flow. Conversely, coastal regions, influenced by sea level variability, exhibited increasing saturation trends. The spatial coherence between groundwater elevation and surface water stages across the study area emphasizes the interconnectedness of these systems. However, the variability in drivers such as canal operation upstream and sea level rise downstream reflects the diverse mechanisms influencing groundwater trends. These mechanisms will need to be monitored as Everglades Restoration proceeds, especially since the strong linear relationship in northern ENP with surface stage in southern 3A will likely change as flows increase into the ENP and surface water stages in 3A decrease.

In Florida Bay, surface water stage dynamics revealed a shift in hydrological patterns compared to WCAs 3A, 3B, and ENP, with significant long-term changes attributed primarily to sea level, which was the most dominant driver of variability. While creek discharge and canal flows contribute to the hydrological regime of the bay, their influence is secondary to the impact of sea level variability. These findings have implications beyond hydrological records by distinguishing where groundwater conditions have operational influence from where they are being shaped by external, sea level forcing. This distinction is relevant to how and where water management can support recovery from dry-season and drought-driven declines or how water management planning may mitigate for sea level rise in the coastal zone. Continued monitoring of hydrological connectivity, groundwater trends, and surface water variability is essential to understand the effectiveness of restoration efforts in this ecologically sensitive and complex landscape.

## Data Availability

Data used in this analysis was primarily sourced from open-access datasets. The public links to access these data sources are as follows: Rainfall, surface/canal water stage, groundwater elevation and creek discharge were accessed from DBHYDRO and USGS using dbhydroR ([https://github.com/ropensci/dbhydroR](https:/github.com/ropensci/dbhydroR)) and UGSG dataRetrieval packages ([https://code.usgs.gov/water/dataRetrieval/-/tree/2.7.12](https:/code.usgs.gov/water/dataRetrieval/-/tree/2.7.12)). Evapotranspiration and Digital Elevation Model is sourced from the Everglades Depth Estimation Network (EDEN) ([https://sofia.usgs.gov/eden](https:/sofia.usgs.gov/eden)) Station locations are sourced from: ([https://sfwmd.dataone.org/view/urn%3Auuid%3Af644085a-437a-4b72-b76e-2f5e2343838b](https:/sfwmd.dataone.org/view/urn%3Auuid%3Af644085a-437a-4b72-b76e-2f5e2343838b)). Tide gage sea level data was obtained from Permanent Service for Mean Sea Level (PSMSL): [https://psmsl.org](https:/psmsl.org)
